# Double Thionated Pyrimidine Nucleobases: Molecular Tools with Tunable Photoproperties

**DOI:** 10.1021/jacs.2c12061

**Published:** 2023-05-25

**Authors:** Abed Mohamadzade, Artur Nenov, Marco Garavelli, Irene Conti, Susanne Ullrich

**Affiliations:** † Department of Physics and Astronomy, University of Georgia, Athens, Georgia 30602, United States; ‡ Dipartimento di Chimica Industriale “Toso Montanari”, Università di Bologna, 40136 Bologna, Italy

## Abstract

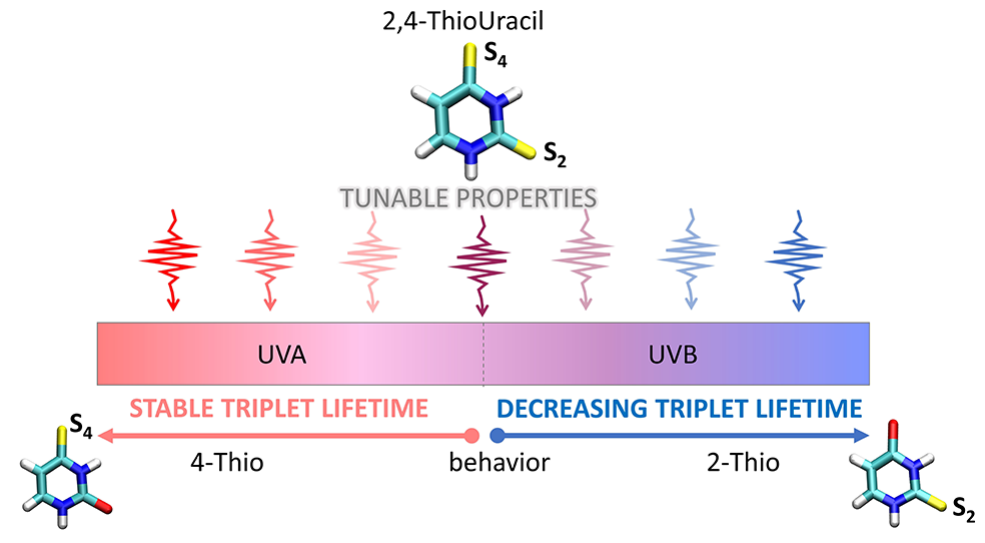

Sulfur-substituted nucleobases are DNA and RNA base derivatives that exhibit extremely efficient photoinduced intersystem crossing (ISC) dynamics into the lowest-energy triplet state. The long-lived and reactive triplet states of sulfur-substituted nucleobases are crucial due to their wide range of potential applications in medicine, structural biology, and the development of organic light-emitting diodes (OLEDs) and other emerging technologies. However, a comprehensive understanding of non-negligible wavelength-dependent changes in the internal conversion (IC) and ISC events is still lacking. Here, we study the underlying mechanism using joint experimental gas-phase time-resolved photoelectron spectroscopy (TRPES) and theoretical quantum chemistry methods. We combine 2,4-dithiouracil (2,4-DTU) TRPES experimental data with computational analysis of the different photodecay processes, which are induced by increasing excitation energies along the entire linear absorption (LA) ultraviolet (UV) spectrum. Our results show how the double-thionated uracil (U), i.e., 2,4-DTU, appears as a versatile photoactivatable instrument. Multiple decay processes can be initiated with different ISC rates or triplet-state lifetimes that resemble the distinctive behavior of the singly substituted 2- or 4-thiouracil (2-TU or 4-TU). We obtained a clear partition of the LA spectrum based on the dominant photoinduced process. Our work clarifies the reasons behind the wavelength-dependent changes in the IC, ISC, and triplet-state lifetimes in doubly thionated U, becoming a biological system of utmost importance for wavelength-controlled applications. These mechanistic details and photoproperties are transferable to closely related molecular systems such as thionated thymines.

## 1 Introduction

Thiobases, sulfur-substituted nucleobase analogs, show entirely different photophysical properties to the canonical bases despite having a very similar chemical structure.^1^ Instead of high photostability due to ultrafast internal conversion (IC),^2−7^ they exhibit characteristic intersystem crossing (ISC) processes with high yields of triplet states. These have been extensively investigated experimentally^2,8−17^ and theoretically^18−29^ due to a growing interest from an application point of view. Beyond the notable medical benefits as prodrugs in phototherapeutic treatments and as versatile photocrosslinking agents, they also draw attention because of their possible phototoxic effects.^10,24,30−37^ Human cells can incorporate different thiobases into their native DNA, possibly leading to genetic code mutations or cell death upon exposure to ultraviolet A (UV-A) irradiation that would otherwise be non-lethal.^38,39^

The present study focuses on the photophysics of thiouracils, where the sulfur substitution of an exocyclic oxygen atom in the 2- and/or 4-positions results in 2-TU, 4-TU, and 2,4-DTU. While maintaining a similar decay mechanism, thiouracils exhibit different deactivation dynamics compared to canonical bases and other modifications.^8,17,29,40−43^ By an ultrafast IC from a photoexcited singlet ^1^ππ* state, the molecule decays to the lowest singlet of ^1^nπ* character, which acts as a doorway to a long-lived and highly reactive triplet manifold with near-unity quantum yield.^8,9,16,40^ However, the time constants attributed to the ISC process and the lifetimes of the final triplet states strongly depend on the position of thionation, hinting at differences in the topography of the potential energy surfaces (PES), critical points (CP), crossing regions, and spin–orbit couplings (SOC). Previous studies in the gas phase show an ultrafast ISC in 2-TU occurring within a few hundred femtoseconds. An anomalously rapid triplet decay follows this step with tens to hundreds of picosecond timescale, whereas in 4-TU, a slower ISC with picosecond timescales leads to trapping in a long-lived triplet state that survives for longer than several nanoseconds.^9,15^

**Figure 1:**
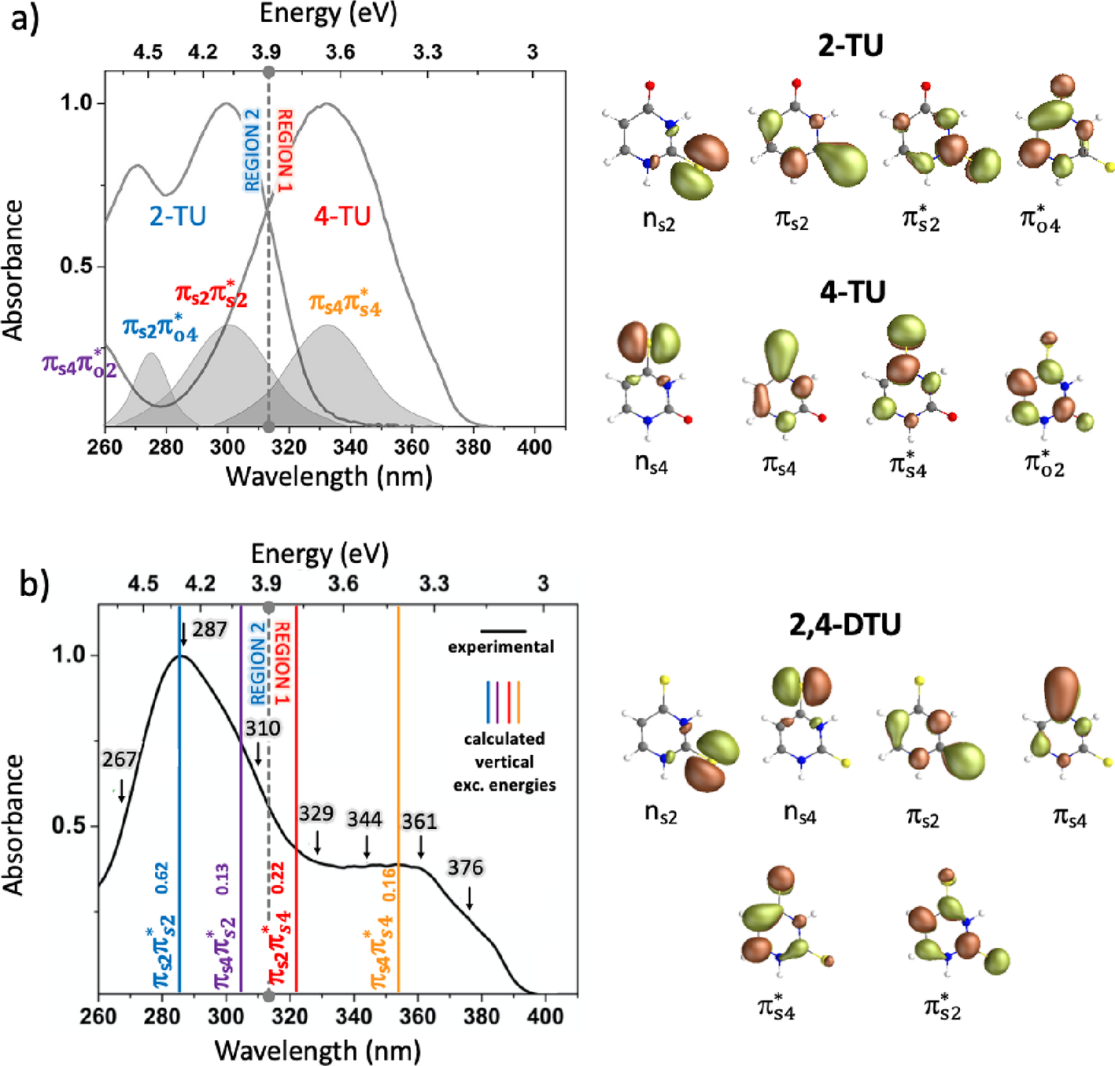
Absorption spectra of (a) 2-TU and 4-TU and (b) 2,4-DTU, all recorded in carbon tetrachloride (CCl_4_) solution, to approximate a gas-phase environment. This choice of non-polar solvent reproduces the shape of the absorption bands observed for gas-phase thiouracils by ref (48) and aligns with gas-phase calculations of transition energies^49^ and simulated absorption spectra.^23,26^ The calculated bright transitions contributing to the absorption bands in (a) are based on refs (9) and (26) in the case of 2-TU and ref (15) for 4-TU. In the spectral window, π_S4_π_S4_^*^ is the only underlying transition for the 4-TU absorption band, while there are two transitions, π_S2_π_O4_^*^ and π_S2_π_S2_^*^, for 2-TU. On the right side of each panel, the natural transition orbitals involved in each excited-state transition are shown, which provide a localized picture of the transition density matrix. The labeling of transitions is chosen to reflect the sulfur 4 and sulfur 2 contributions. In (b), the colored vertical lines on top of the 2,4-DTU absorption spectrum show the MS-CASPT2(18,14)/ANO-RCC calculated vertical excitation energies of the different bright states (see Computational Details section and Figure S1 in the SI). The number associated with each line is the oscillator strength of the corresponding transition. A gray dashed vertical line divides the linear absorption spectra of 2-TU and 4-TU in (a) and 2,4-DTU in (b) into region 1 and region 2. The black arrows represent the position of the pump wavelengths. The 250 nm pump wavelength is not shown due to the cutoff of the experimental absorption spectrum in CCl_4_.

The present investigation into intricate details of the thiouracil photodynamics is motivated by a visual correlation of characteristic LA bands in the spectra of 2-TU, 4-TU, and 2,4-DTU. Specifically, 2-TU and 4-TU show very distinct LA spectra. The former has the maximum absorption peak in the UV-B and the latter in the UV-A window (Figure 1a).^23,26^ 2-TU shows two bands centered around 300 and 270 nm. In contrast, 4-TU has a central peak at around 330 nm, with a blue-shifted second absorption band rising below 280 nm. The nature of the two different bands is the same in both systems, which correspond to^1^ππ* states. The former presents bonding and antibonding orbital components more localized on either sulfur 4 or 2 and, accordingly, is labeled π_S4_ (π_S4_^*^) or π_S2_ (π_S2_^*^) in Figure 1a. Transitions originating from the same bonding orbital but to higher antibonding orbitals contribute to the second band. These orbitals are labeled π_O4_^*^ and π_O2_^*^ for 2-TU and 4-TU, respectively, but lack localization.

Intriguingly, the nature of the four orbital transitions contributing to the 2,4-DTU absorption spectrum can be directly correlated with sulfur 2 and 4 localized transitions of the singly substituted thiouracils (see Figure 1), even though the original order changes upon thionation of the second oxygen. The corresponding intensities are also modified as a result of interactions between the four close-lying excited states. Mostly, this induces a red shift of the two 4-TU bands π_S4_π_S4_^*^ and π_S4_π_o2_^*^. Despite the non-negligible overlap of the transitions, the two prominent absorption bands of 2,4-DTU (Figure 1b) qualitatively coincide with the 4-TU and 2-TU spectra (Figure 1a). Therefore, the LA spectrum of 2,4-DTU can be divided into two different regions as indicated by the gray dashed line crossing Figure 1b: a less intense band covering the UV-A (400 nm–315 nm, labeled as region 1) and a more intense absorption band in the UV-B (315 nm–260 nm, labeled as region 2). While the 2,4-DTU absorption spectrum is not a direct linear combination of the absorption spectra of 2-TU and 4-TU, qualitative resemblances of absorption bands in the two regions are observed. Therefore, from a simplistic point of view, one might hypothesize that some characteristics of 2-TU and 4-TU are preserved upon doubling thionation. This would suggest that the photoexcitation of 2,4-DTU with UV-A light would result in photophysics similar to that of 4-TU, whereas excitation with UV-B would resemble the 2-TU photophysics. If this is indeed the case, 2,4-DTU can serve as a tunable photosensitizer with a wavelength-dependent triplet quenching rate, thus allowing versatility in various medical therapy perspectives. By side-stepping Kasha’s rule, it can also contribute to the development of small and bio-compatible triplet emitters of particular interest in organic light-emitting diodes (OLEDs), which are emerging in ever more low-level lighting applications.^44−47^

Hence, the present joint experimental and theoretical gas-phase TPRES study aims at scrutinizing the proposed hypothesis, i.e., (i) conservation of the photophysics of the singly substituted uracils in the doubly thionated nucleobase and (ii) dependence on the excitation energy. For this purpose, we comprehensively investigate the dynamic picture of the ultrafast relaxation mechanisms of 2,4-DTU from the UV-A to UV-B window. Eight different wavelengths are selected across the LA spectrum and are shown by black arrows in Figure 1b (except for 250 nm, which is beyond the measured LA spectral window). The dynamics of the molecule are measured experimentally at each wavelength and are backed up and further explained by quantum chemical computations. We reveal the different decay paths that contribute to the relaxation mechanism of 2,4-DTU when photoexcited across the LA spectrum. The adopted high-level CASPT2 multireference perturbatively corrected method also supports the TRPES signals by providing ionization potentials (IPs), oscillation strengths, and minimum energy paths, thus validating the theoretical model and proposed decay routes.

## 2 Results

The peaks observed in the two distinct absorption regions of 2,4-DTU (region 1 and region 2 in Figure 1b) match very well with the calculated energies of the four bright ^1^ππ* states, represented as solid vertical lines in Figure 1b. These four ^1^ππ* states resemble, respectively, the nature of the two ^1^ππ* states localized on the singly substituted 4-TU and 2-TU systems described above. This becomes evident when comparing the orbitals of 2-TU and 4-TU with the ones of the doubly thionated 2,4-DTU. The ^1^π_S4_π_S4_^*^ transition (orange line) is mainly responsible for the low energy band (region 1), although the higher-lying states ^1^π_S2_π_S4_^*^ and ^1^π_S4_π_S2_^*^ (red and purple lines, respectively) contribute, to some extent, toward shorter wavelengths.^26^ On the other hand, the more intense band in region 2 involves complex contributions from the three higher bright transitions, ^1^π_S2_π_S4_^*^, ^1^π_S4_π_S2_^*^, and ^1^π_S2_π_S2_^*^ (red, purple, and blue lines, Figure 1b). These ^1^ππ* states (^1^π_S4_π_S4_^*^, ^1^π_S2_π_S4_^*^, ^1^π_S4_π_S2_^*^, ^1^π_S2_π_S2_^*^) resemble the nature of the singly substituted 4-TU and 2-TU derivatives, characterized by comparable electronic transitions (see orbitals in Figure 1a,b).

The contribution of the four dark ^1^*n*π* states in the LA spectrum can be ignored due to their negligible intensity and close-to-zero oscillator strength.^26^ However, their primary role is in the relaxation dynamics of the excited states.

The high density of low-lying electronic states, characterized by four bright ^1^ππ* states and four additional dark ^1^*n*π* states (eight in total, documented in Table S1),^26,40,50^ may render the 2,4-DTU mechanism more complicated than simple competition between pathways of the singly substituted thiouracils. The higher number of available states along the relaxation path might cause the mixing and crossing of pathways in an unpredictable way.^8,20,40,49^

### 2.1 Excited-State Lifetimes

**Chart 1:**
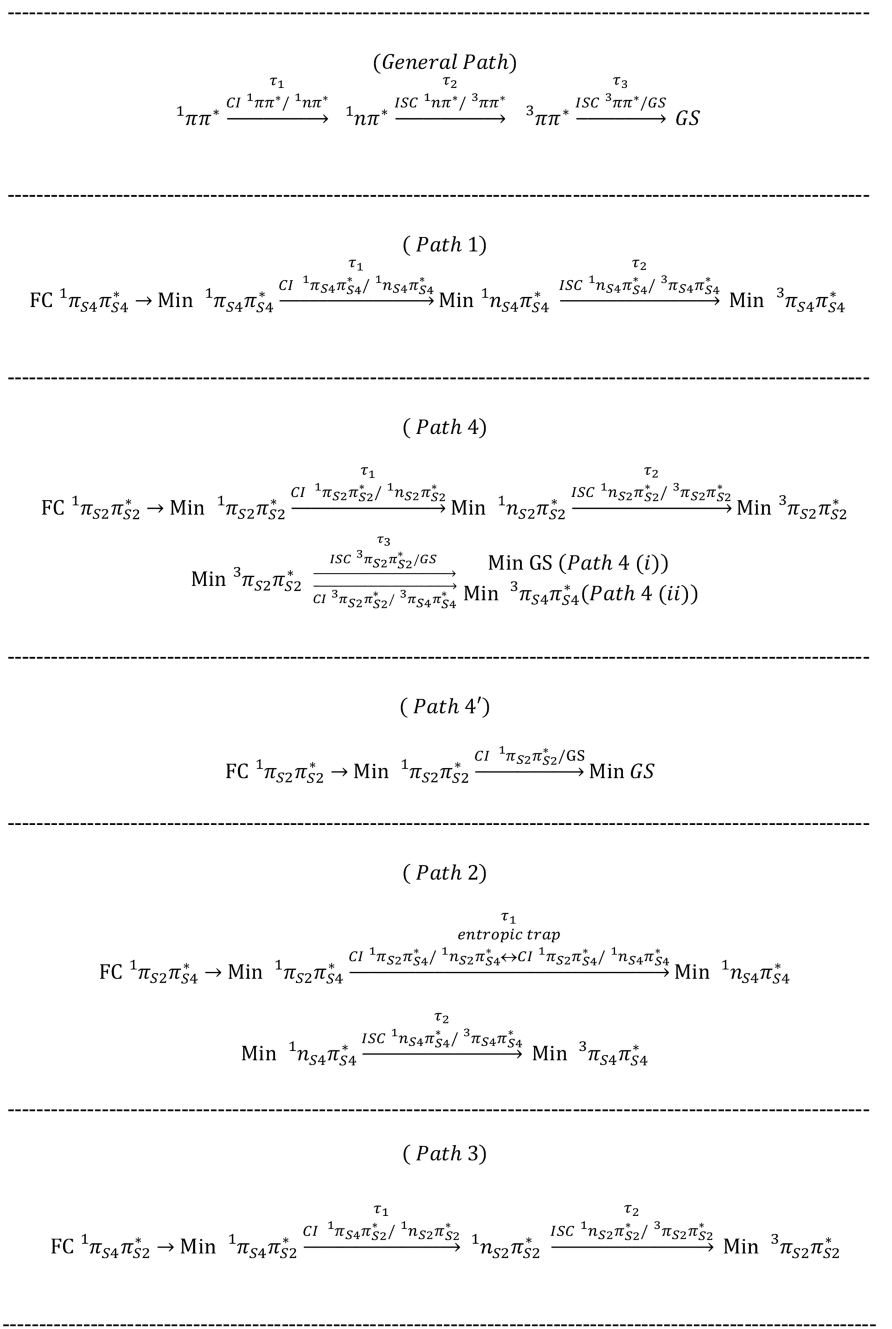
Different Decay Paths

Briefly, the overall dynamics of the thiouracils can be summarized by an ultrafast IC from the photoexcited singlet ^1^ππ* state to the lowest singlet of ^1^*n*π* character (lifetime τ_1_), followed by an ISC process (τ_2_) that efficiently leads into the lowest triplet state, which survives until the final GS recovery (τ_3_) (see Chart 1)

The lifetimes associated with each process (τ_1_, τ_2_, and τ_3_) strongly depend on the position and degree of substitution (sulfur 2 or sulfur 4 or both) and on the pump wavelength that defines the photoexcited band of ππ_*S*4_^*^ or ππ_*S*2_^*^ character (see Figure 1), for example, a fast ISC in and out of the triplet state in 2-TU and a slow ISC rate and a long-lived triplet state in 4-TU.^8,9,15^ Previous gas phase measurements across the first absorption band of 2,4-DTU hint at 4-TU-like ISC dynamics. Considering these similarities, it is logical to systematically explore whether the photophysics of singly substituted bases in 2,4-DTU is conserved across the UV-A and UV-B regions of the absorption spectrum.

**Figure 2:**
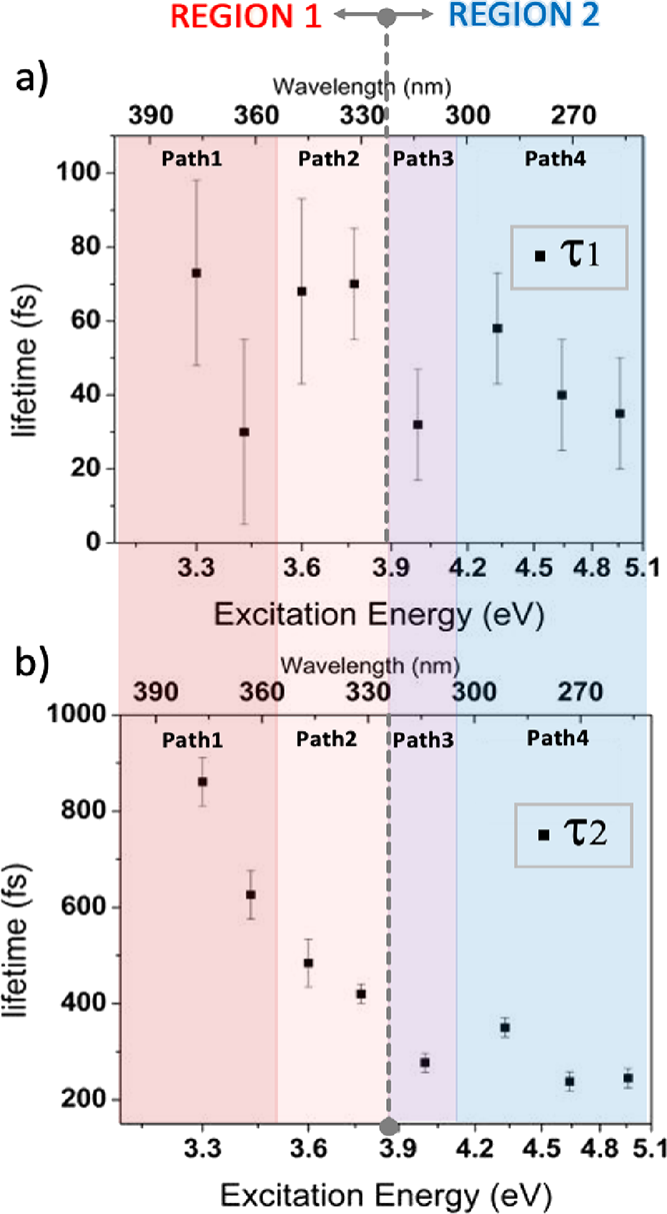
Lifetime versus the excitation energy (wavelength) plots of the first (τ_1_, top) and second (τ_2_, bottom) time constants in the decay model for all the excitation wavelengths documented in Table 1 (376, 361, 344, 329, 310, 287, 267, and 250 nm). Each colored region groups excitation energies with the same type of calculated decay path (from Path 1 to Path 4). The same colors are adopted in Table 1. The 376, 361, and 344 nm data are taken from ref (8).

For all wavelengths shown in black arrows across the spectral window in Figure 1b, the 2,4-DTU TRPES are best described by a kinetic model consisting of a sequential triple exponential decay. Again, these time constants characterize the IC (τ_1_), ISC (τ_2_), and the final triplet lifetime (τ_3_) (see Table 1 and Figure 2).^8,9,15,16,21,26,29,40^ Global lifetime analysis of the TRPES signal simultaneously extracts the evolution-associated spectra (EAS) and corresponding kinetic fit components. The fitting function for the sequential triple decay is documented in Section S4.2 of the Supporting Information (SI), and the fitted time traces of the integrated signals with contributing components are presented in Figure S6 of the SI. Although more than one transition may be excited simultaneously when the pump wavelength is tuned across the intermediate region of the absorption spectrum, the same sequential kinetic model is used to extract the dynamics. Using multiple sequential decays would overparameterize the fit. The time constants and EAS of the parallel decay paths are too similar and cannot be disentangled in the global analysis. Instead, an averaged time constant and EAS are extracted that are weighted by the individual contributions of the parallel paths. Fits with only two exponential decay components are presented in Section S4.1 of the SI and show the model’s inadequacy.

**Table 1:** Decay Constants for the Individual Deactivation Steps Derived from Global Lifetime Analysis of the TRPES Spectra and Fitting a Triple Sequential Exponential Decay Model to the Integrated Time Traces^a^

wavelength (nm)	energy (eV)	τ_1_ (fs)	τ_2_ (fs)	τ_3_ (ns)
376	3.30	73 ± 25	861 ± 50	>2
361	3.43	30 ± 25	626 ± 50	>2
344	3.60	68 ± 25	484 ± 50	>2
329	3.77	70 ± 20	420 ± 20	>2
310	4.00	32 ± 20	277 ± 20	>1.5
287	4.33	58 ± 20	350 ± 20	>1
267	4.64	40 ± 20	240 ± 20	0.54
250	4.96	35 ± 20	245 ± 20	0.33

a While τ_1_ and τ_2_ are extracted from a global fit to the short-range region (−1 to 3.5 ps), keeping the long time constant fixed, τ_3_ is extracted from a fit on the energy integrated long-range TRPES up to 300 ps. The error associated with each time constant is based on the uncertainty in the reproducibility of results across multiple TRPES experiments.

All the exponential decay constants are listed in Table 1 and visualized in Figure 2. Typically, a decrease in the state’s lifetime with an increase in the excitation energy is expected for relaxation pathways with a barrier because excess energy will ease barrier crossing.^51^ Furthermore, specific to ISC, molecular distortions can increase the SOC between the singlet and triplet states and lead to a systematic enhancement in the ISC rate constant. For example, out-of-plane vibrations have been shown to leave the energy gap between S_1_ and T_1_ states nearly unchanged but increase their mutual SOC substantially by mixing some σ or *n* and π* characters into the electronic wavefunctions.^52,53^Figure 2 shows the first two time constants (panels a and b, respectively) as a function of the excitation wavelength. The absence of a systematic trend is more evident for τ_1_ (panel a) but also for τ_2_ (see outlier at 287 nm in panel b). This irregular pattern in time constants supports the idea of different relaxation paths at higher excitation energies. The primary goal of the present work is to characterize the underlying pathways and explain the observed irregular pattern in experimental decay constants.

### 2.2 TRPES Spectra and Associated Decay Paths

**Figure 3:**
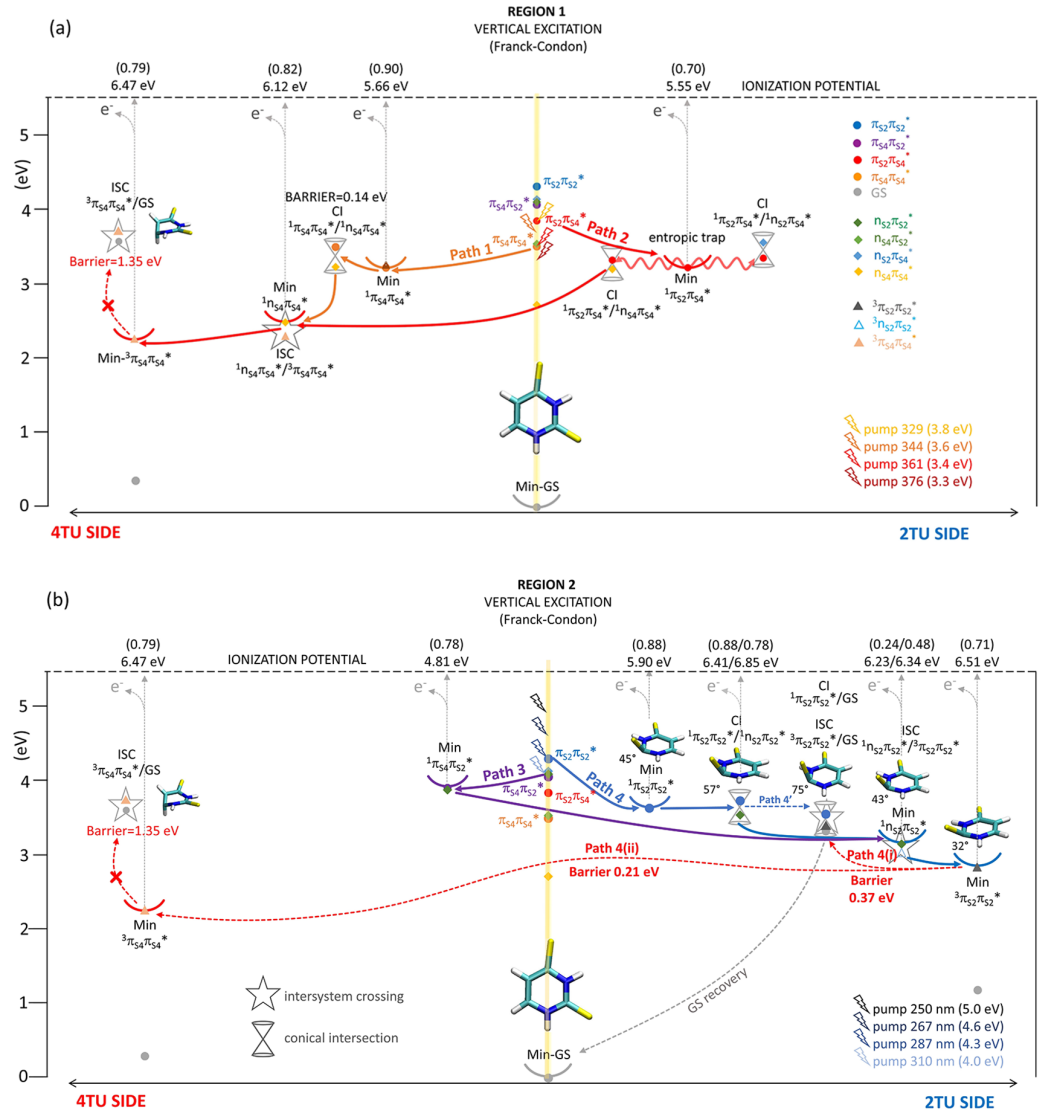
(a) CASPT2-calculated decay paths for excitations at the four lowest pump energies adopted in the TRPES experiments: 376, 361, 344, and 329 nm. The corresponding experimental time constants are reported in the first four rows of Table 1. (b) Calculated relaxation routes upon excitations at 310, 287, 267, and 250 nm. The corresponding experimental time constants are reported in the last four rows of Table 1. The ionization energies for ionization from the excited state and the Dyson amplitudes (intensities, numbers in parenthesis) are reported on top of each critical point, above the dashed horizontal line.

To discover the reason behind the observed discontinuity in the time constants, we computationally analyzed the 2,4-DTU decay paths. Four major decay paths, starting from each bright ^1^ππ* state in the UV-A–UV-B regions, are characterized and labeled as Path 1, Path 2, Path 3, and Path 4. Each Path has been tracked by characterizing all the CPs and the lowest conical intersection (CI) and ISC regions populated along the relaxation routes (see Figure 3). Based on the eight different pump wavelengths from 376 to 250 nm adopted in the TRPES experiment, we hypothesized which pathways will be mainly or simultaneously populated and how the properties of the decay paths smoothly change by moving from the longest to the shortest wavelength.

Here, we describe the induced decay paths for each adopted pump wavelength that lies on the LA spectrum (black arrows in Figure 1b).

**Figure 4:**
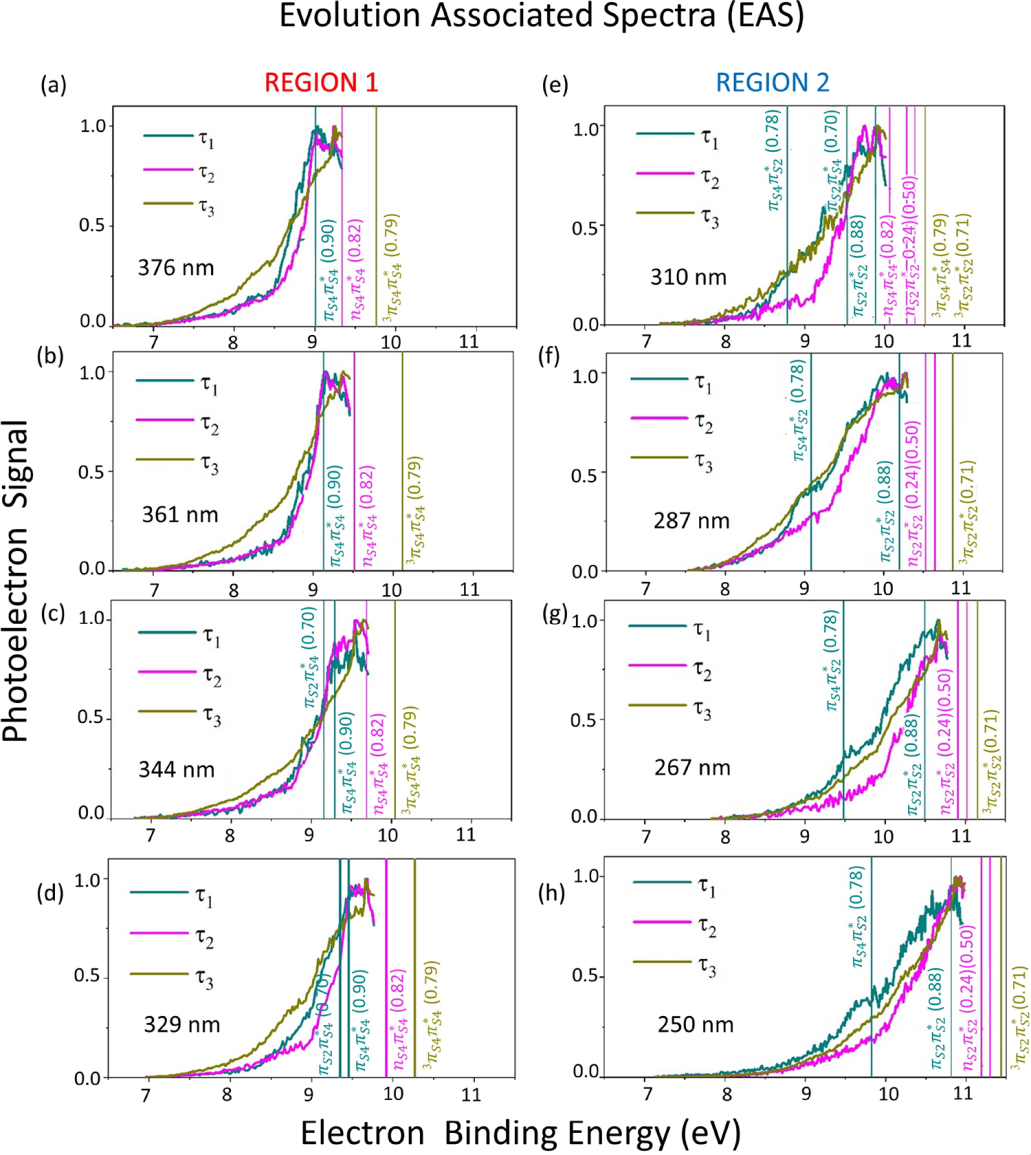
Evolution-associated spectra (EAS) are extracted from the global lifetime analysis of the experimental TRPES (Figure S3, SI). The EAS are normalized and presented in the order of decreasing excitation wavelength, including the first three from our previous study.^8^ The left column refers to excitation energies in region 1, and the right column refers to region 2. In the EAS panels, we show the estimated BEs (vertical lines, Table S2 in the SI) and the corresponding intensities (Dyson norm values in parenthesis, Figure 3a,b). If two Dyson norms are addressed to the same vertical line, two close BEs exist for the same critical point with non-negligible different Dyson values. Non-normalized EAS are shown in Figure S7. The spectral cutoff of the EAS in the high electron binding energy region is imposed by the total photon energy available in the experiment.

The two extremes of the LA spectrum show minimum overlap with other transitions (see Figure 1b) and are respectively covered by ^1^π_S4_π_S4_^*^ and ^1^π_S2_π_S2_^*^ bands, involving sulfur 4 and sulfur 2 localized electronic transitions. Then, as it will be demonstrated, the decay mechanisms associated with pump pulses centered at the two tails (labeled Path 1 and Path 4) resemble the deactivation in the singly substituted 4- and 2-TU, respectively, and are thus tagged “pure” decay paths. “Hybrid” paths (Path 2 and Path 3) arise from pump pulses centered in the LA spectrum’s intermediate region. In this region, the overlapping bands lead to a simultaneous population of more than one bright state and a complex mixture of the 4- and 2-TU characteristics. Depending on the anticipated Path, the eight adopted pump pulses are grouped in colored areas in Table 1 and Figure 2. In the next section, characteristic CPs along each Path are discussed in the context of experimental time constants and EASs. The purpose of the normalized EASs in Figure 4 is the identification of spectral features associated with photoionization from CPs. Additional plots of the EASs are provided in Sections S4 and S5 of the SI that highlight other aspects such as relative intensities of EASs, wavelength-dependent spectral shifts due to vibrational excitations, and spectral shifts associated with changes in IPs.

#### 2.2.1 Probing 2-TU and 4-TU Localized Dynamics through “Pure” Decay Paths

##### Path 1

The calculated vertical lines in Figure 1b indicate the maximum of each ^1^ππ* absorbing band. The two lowest 376 and 361 nm excitation energies, black arrows in the UV-A region 1 in Figure 1b, lie below the orange vertical line representing the first ^1^π_S4_π_S4_^*^ band’s maximum. Therefore, we can postulate with high certainty that these two pump wavelengths populate exclusively the corresponding lowest ^1^π_S4_π_S4_^*^ state. The calculated minimum energy path involving all the characterized CPs, CIs, and ISCs, labeled Path 1, starts from the orange arrow in Figure 3a. Upon relaxation on the PES of the ^1^π_S4_π_S4_^*^ state, the system has access to the ^1^π_S4_π_S4_^*^/^1^*n*_S4_π_S4_^*^ crossing region (labeled CI-^1^π_S4_π_S4_^*^/^1^*n*_S4_π_S4_^*^) placed just 0.14 eV above the ^1^π_S4_π_S4_^*^ minimum, Min-^1^π_S4_π_S4_^*^. From the CI, the system can reach the ^1^*n*_S4_π_S4_^*^ minimum (Min-^1^*n*_S4_π_S4_^*^), where an ISC process (ISC-^1^*n*_S4_π_S4_^*^/^3^π_S4_π_S4_^*^) facilitates the decay to the minimum of the lowest triplet state of ^3^π_S4_π_S4_^*^ nature (Min-^3^π_S4_π_S4_^*^). All along the entire decay route, the molecule remains roughly planar. Path 1 can be summarized in Chart 1.

This photoinduced deactivation process, which involves orbitals localized on sulfur 4, strongly resembles the previously documented decay path in singly substituted 4-TU.^15−17^ The study attributed τ_2_ and τ_3_ to the ISC and the final triplet-state lifetimes with picosecond and nanosecond timescales, respectively,^15^ and matches the observation here for 2,4-DTU (see first two rows of Table 1). Also, see Table S3 in Section S8 of the SI for a comparison between 2,4-DTU and 4-TU decay constants. The typical nanoseconds constant (τ_3_) of the triplet manifolds suggests the absence of any accessible decay funnel that facilitates GS recovery. Indeed, the ISC crossing point between the triplet state and the GS is 1.35 eV higher in energy relative to the final Min-^3^π_S4_π_S4_^*^ for 2,4-DTU (see ISC-^3^π_S4_π_S4_^*^/GS in Figure 3a). This justifies the long-lived triplet state and predicts radiative relaxation as an alternative decay route at extended times.

The decrease in the τ_1_ IC lifetime from 376 to 361 nm (Figure 2 and Table 1) is explained by excess vibrational energy upon higher excitation energy that facilitates the barrier crossing. However, the ISC process involving Min-^1^*n*_S4_π_S4_^*^ and^3^π_S4_π_S4_^*^ is nearly barrierless. Because out-of-plane molecular distortion modes can enhance the ISC process,^52−54^ the faster ISC rate (τ_2_) can be attributed to random out-of-plane modes, activated by increasing excitation energy, while an in-plane geometry still governs the dominant pathway.

The calculated Path 1 and the corresponding experimental lifetimes are supported by a direct comparison of experimental EAS (Figure 4a,b) and calculated electron binding energy (BE) values computed for CPs along Path 1. See Table S2 in the SI for BE estimations based on the ionization energies, the vibrational energy gain during electronic relaxation, and the propensity for ionization into a vibrationally excited cationic state. The lowest IP at each critical geometry visited along the decay route is shown on top of the dashed line in Figure 3a. The obtained BE values, evaluated at Min-^1^π_S4_π_S4_^*^ and Min-^1^*n*_S4_π_S4_^*^ (blue and magenta vertical lines in Figure 4a,b), match the τ_1_ and τ_2_ peaks of the associated spectra (blue and magenta bands), but for the latter, the maximum signal of Min-^1^*n*_S4_π_S4_^*^ is predicted slightly beyond the experiment observation window. The experimental EAS signals recorded at 376 and 361 nm wavelengths show a cutoff at ∼9.5 eV. Similar to the ^1^*n*_S4_π_S4_^*^ EAS, this partially crops out the maximum of the triplet ^3^π_S4_π_S4_^*^ signal. However, by partially capturing these spectra, the state assignments are possible by comparing the features, onsets, and relative shifts of spectral components and taking the extracted decay constants into account (see Section S5.2 of SI).

Comparing the onset of the EAS for the three different components in Figure 4a,b leads to the observation that the onset of all spectra rises at similar energies (around 7.5 eV) despite higher BE estimates for ionization from the ^1^*n*_S4_π_S4_^*^ singlet (τ_2_ EAS) and even more the ^3^π_S4_π_S4_^*^ triplet minima (τ_3_ EAS) compared to the ^1^π_S4_π_S4_^*^ (τ_1_ EAS). The observed onsets fall within the range of the adiabatic IP of 2,4-DTU. Normalization of the EASs gives a false impression of the actual signal amplitudes of the τ_1_, τ_2_, and τ_3_ spectra in the 7.5–8.5 eV range. Instead, the relative signal intensities are more accurately represented in the non-normalized EAS plots in Figure S7. The ^1^π_S4_π_S4_^*^ signal dominates the low-BE region in agreement with the predicted higher IPs of the ^1^*n*_S4_π_S4_^*^ and ^3^π_S4_π_S4_^*^ minima. In fact, if a Δν = o propensity is assumed for the photoionization step (BEs reported in Figure 3), the band maxima of the τ_2_ and τ_3_ EASs shift beyond the observable energy range. This high BE cutoff of the EASs is associated with the total photon energy used for the TRPES experiments.

##### Path 4

Turning our attention to the UV-B extreme of the LA spectrum in region 2, we can speculate that the three shortest pump energies, i.e., 287, 267, and 250 nm, will dominantly populate the highest bright state ^1^π_S2_π_S2_^*^ (blue vertical line in Figure 1b). The calculations shown in Figure 3b assign the Path 4 decay route to this state*.* From the FC region, following the route starting with the solid blue arrow, the system relaxes along a barrierless path that finally leads to the triplet-state minimum Min-^3^π_S2_π_S2_^*^. This triplet state is located on the right side of Figure 3b and has a distinctive character from the Min-^3^π_S4_π_S4_^*^ on the left side. Specifically, the ^3^π_S2_π_S2_^*^ and ^3^π_S4_π_S4_^*^ triplet minima represent two different valleys on the adiabatic surface of the lowest triplet state and the ^3^π_S4_π_S4_^*^ represents the global minimum. During Path 4, the molecule passes through a ^1^π_S2_π_S2_^*^ →^1^*n*_S2_π_S2_^*^ CI and a ^1^*n*_S2_π_S2_^*^ →^3^π_S2_π_S2_^*^ ISC region. The two crossing regions are characterized by a pronounced out-of-plane bending of sulfur 2, reaching a distortion of around 57°, closely resembling the corresponding decay mechanism of 2-TU from its second ^1^π_S2_π_S2_^*^ bright state.^9,40^ Using similar reasoning as above for Path 1, an increased excitation of out-of-plane molecular distortions is expected to shorten ISC times.^52−56^ Along Path 4, excess pump photon energy can be deposited into the sulfur 2 bending mode to ease access to the crossing region, which is located along this coordinate and promote ISC. This effect can explain the significantly shorter ISC time constants, τ_2_, in Path 4 compared to Path 1 and the trend observed for the τ_2_ time constants associated with Path 4. However, there is also a clear discontinuity in the τ_2_ pattern with respect to Path 3, which is discussed further in the Path 3 section below.

Two feasible options can be considered for the depopulation of Min-^3^π_S2_π_S2_^*^ (depicted by red dashed arrows in Figure 3b): Path 4, (i) the system relaxes to the GS through an accessible ISC region with the GS (ISC-^3^π_S2_π_S2_^*^/GS), characterized by an even more considerable sulfur 2 bending (∼75°, see Figure 3b). This path follows the red dashed line to ISC-^3^π_S2_π_S2_^*^/GS region and then the gray dashed line for GS recovery. A similar ISC pathway back to the GS was previously characterized in the singly substituted 2-TU system^9,40^ and resulted in a shortening of the triplet-state lifetime. Remarkably, we note a nearly threefold decrease in the τ_2_ lifetimes of this Path compared to Path 1, confirming the resemblance with the fast ISC process of the singly substituted 2-TU. A direct comparison between the 2,4-DTU and 2-TU decay constants is made in Table S3 of the SI.

Alternatively, along Path 4 (ii), the deactivation can occur through an adiabatic process that leads to the near-planar global triplet minimum Min-^3^π_S4_π_S4_^*^, from where the GS recovery should take place on timescales exceeding nanoseconds, similar to the τ_3_ in Path 1 (see Table 1). However, considering the short experimental time constants (see τ_3_ in the last three rows of Table 1), this path is less favorable.

Thus, Path 4 is summarized in Chart 1.

On a side note, we outline another viable path for depopulating ^1^π_S2_π_S2_^*^, labeled Path 4*′*. This path is shown with a dashed blue line in Figure 3b and leads to an accelerated GS recovery, highlighted by the gray dashed arrow. This Path, activated along the sulfur 2 bending mode, needs to overpass the first CI-^1^π_S2_π_S2_^*^/^1^*n*_S2_π_S2_^*^ crossing region to reach the second CI with the GS (CI-^1^π_S2_π_S2_^*^/GS). The CI-^1^π_S2_π_S2_^*^/GS lies in the same region as the above-described ISC-^3^π_S2_π_S2_^*^/GS. As the excitation energy increases, an increase in the population of Path 4′ should induce a lower triplet-state quantum yield. For 2,4-DTU, such a decrease in the triplet-state quantum yield as a function of photoexcitation energy has not previously been investigated either theoretically or experimentally. However, CI-^1^π_S2_π_S2_^*^/GS is considered an unlikely path due to the efficient depopulation of the ^1^π_S2_π_S2_^*^ state through the CI-^1^π_S2_π_S2_^*^/^1^*n*_S2_π_S2_^*^ decay funnel prior to reaching the larger sulfur 2 bending that is required for the CI-^1^π_S2_π_S2_^*^/GS IC (75° compared to 57°). This secondary Path 4′ can be summarized in Chart 1.

The experimental EAS and calculated BEs support the Path 4 population upon excitation at 287, 267, and 250 nm (Figure 4f, g, and h, respectively). The three corresponding photoelectron signals show their τ_1_ maxima (blue peak) at energies well-matching with the calculated BEs at Min-^1^π_S2_π_S2_^*^. The apparent shoulder (bump) on the left side of the blue band (τ_1_) matches the calculated BE at Min-^1^π_S4_π_S2_^*^. This indicates a minor population of Path 3 that arises from the low-lying ^1^π_S4_π_S2_^*^ band (see Figure 1b) and is discussed in the next section. The ^1^*n*_S2_π_S2_^*^ and triplet ^3^π_S2_π_S2_^*^ BE signals are assigned to the τ_2_ and τ_3_ bands; however, their maxima are not fully captured by the total photon energy. Nevertheless, they show similar spectral features for 287, 267, and 250 nm (see Section S5.2 of SI for the direct comparison of normalized EAS).

In summary, Path 1 and Path 4 relaxation processes arise from electronic transitions mainly localized on sulfur 4 and 2, respectively. The relaxation dynamics initiated by pumping at the extremes of the LA spectrum present similarities to the corresponding singly substituted compounds: 4-TU, as in Path 1, leads to a stable ^3^π_S4_π_S4_^*^ long-lived triplet minimum, from where decay funnels to the GS are not accessible due to the high energy barrier. In contrast, 2-TU, as in Path 4, shows faster ISC processes (τ_2_) followed by a shorter triplet lifetime (τ_3_) due to accessible ISC regions lying along the sulfur 2 bending mode (see Table S3 in the SI). These gas-phase mechanisms can be extended to a polar environment, such as water (see further computational details in the SI, Section S7), and consequently also to scenarios of relevance to biological applications.

#### 2.2.2 Probing the Mixed Dynamics through The “Hybrid” Decay Paths

Photoexcitation at the intermediate wavelengths across the 2,4-DTU LA spectrum (i.e., between 344 and 310 nm) leads to a mixed population of the two close-lying electronic states ^1^π_S2_π_S4_^*^ and ^1^π_S4_π_S2_^*^. The degree of contribution of each state to the photoinduced dynamics depends on the pump wavelength (see Figure 1b). These intermediate decay paths, which mainly originate from ^1^π_S2_π_S4_^*^ and ^1^π_S4_π_S2_^*^ states, are labeled as Path 2 and Path 3 and mix the characteristics of the singly substituted 4-TU or 2-TU. Thus, they exhibit “hybrid” decay routes, as opposed to the 4-TU-like or 2-TU-like paths (tagged “pure” routes) observed upon excitation to ^1^π_S4_π_S4_^*^ and ^1^π_S2_π_S2_^*^, as discussed in the previous section.

##### Path 2

Based on the LA spectrum in Figure 1b, 344 and 329 nm pump pulses are located between the calculated vertical excitation energies of the ^1^π_S4_π_S4_^*^ and ^1^π_S2_π_S4_^*^ states (orange and red vertical lines). We estimate that these two wavelengths induce the simultaneous activation of Path 1 and Path 2. In the initial relaxation of Path 2 (red arrow in Figure 3a), the ^1^π_S2_π_S4_^*^ minimum works as an entropic trap. The entropic trap represents a flat region on the PES (wavy red line) where the system couples non-adiabatically to the ^1^*n*_S2_π_S4_^*^ and ^1^*n*_S4_π_S4_^*^ state through close-lying CIs (CI-^1^π_S2_π_S4_^*^/^1^*n*_S2_π_S4_^*^ and CI-^1^π_S2_π_S4_^*^/^1^*n*_S4_π_S4_^*^, both energetically accessible). However, calculations cannot identify a crossing structure in the proximity of the ^1^*n*_S2_π_S4_^*^ minimum, leading to the conclusion that the decay route through CI-^1^π_S2_π_S4_^*^/^1^*n*_S2_π_S4_^*^ does not facilitate further energy relaxation. The only escape route is relaxation through the CI-^1^π_S2_π_S4_^*^/^1^*n*_S4_π_S4_^*^ to ^1^*n*_S4_π_S4_^*^ minimum (Min-^1^*n*_S4_π_S4_^*^) from where the system follows the same fate as Path 1, eventually decaying to the ^3^π_S4_π_S4_^*^ triplet minimum*.* As seen in Table 1 and Figure 2, τ_1_ does not decrease as expected for pump wavelengths from 344 toward 329 nm. This deviation from the expected trend is attributed to the role of the entropic trap along Path 2, where multiple isoenergetic states detain the molecule for a longer time before eventually finding the decay funnel. Path 2 is summarized in Chart 1.

Experimental EAS in Figures 4c,d show two features contributing to τ_1_. They are assigned to the involvement of ^1^π_S2_π_S4_^*^ and ^1^π_S4_π_S4_^*^ based on the calculated BEs. Min-^1^π_S2_π_S4_^*^ exhibits a slightly lower BE value and a lower intensity compared to Min-^1^π_S4_π_S4_^*^ (vertical blue lines in Figure 4c,d). The contribution from both ionization bands causes a broader τ_1_ EAS compared to the pure Path 1, which involves only the ^1^π_S4_π_S4_^*^ (Figure 4a,b). Another factor that confirms the involvement of ^1^π_S2_π_S4_^*^ is the lower energy onset of the τ_1_ EAS. The amplitude of this signal increases from Figure 4c to d, indicating an increasing contribution of this state. The relative structures of τ_2_ and τ_3_ EAS do not change significantly to those observed at excitations λ > 344 nm, which supports the interpretation that the decay follows Path 1 for the subsequent ISC dynamics at longer times.

##### Path 3

The pump pulse centered at 310 nm matches roughly with the calculated ^1^π_S4_π_S2_^*^ vertical excitation energy (purple vertical line in Figure 1b). This implies the dominance of the associated decay path labeled Path 3. The ^1^π_S4_π_S2_^*^ state degenerates with the ^1^*n*_S2_π_S2_^*^ state (Figure 3b), which allows for a barrierless and, thus, ultrafast IC process. From there, the system continues to relax on the ^1^*n*_S2_π_S2_^*^ state through sulfur 2 out-of-plane bending motion and finally merges into Path 4, described earlier. Path 3 is summarized in Chart 1.

The absence of barriers or entropic traps suggests overall fast IC and ISC processes, corresponding to significantly shortened lifetimes of τ_1_ = 32 fs and τ_2_ = 277 fs (see Figure 2) compared to 329 nm (Path 2) and 287 nm (Path 4) excitations. The observation of longer time constants at the shorter excitation wavelength may be unexpected and therefore requires discussion. Path 4 and Path 3 are launched from almost planar FC geometries of ^1^π_S2_π_S2_^*^ and ^1^π_S4_π_S2_^*^, respectively, and require sulfur 2 out-of-plane bending. Even if the IC process (τ_1_) for both paths is barrierless, Path 4 approaches the CI-^1^π_S2_π_S2_^*^/^1^*n*_S2_π_S2_^*^ from Min-^1^π_S2_π_S2_^*^ along a flatter decay path that is accompanied by a large sulfur 2 bending motion. In contrast, a continued gradient in Path 3 drives the motion through CI-^1^π_S4_π_S2_^*^/^1^*n*_S2_π_S2_^*^ and along a coordinate with smaller sulfur 2 out-of-plane distortion. Combined, these effects lead to slightly faster IC dynamics along Path 3. Then, in a sequential decay model, the fast-driven depopulation of the initially excited state (^1^π_S4_π_S2_^*^) continues to speed up the population dynamics of subsequent states, including the ISC dynamics characterized by the time constant τ_2._

According to the computational results, 310 nm is the longest excitation wavelength that allows access to the energetically higher-lying valley on the lowest triplet state (Min-^3^π_S2_π_S2_^*^ in Figure 3b). Thus, 310 nm represents the predicted turning point in the 2,4-DTU behavior where depletion of the triplet manifold may exhibit 2-TU characteristics such as sulfur 2 out-of-plane mediated ISC back to the GS. In 2-TU, this mechanism has been associated with a significantly shorter triplet-state lifetime. However, due to the substantial barrier to access the equivalent crossing point in 2,4-DTU, τ_3_ at 310 nm remains long (>1.5 ns). A notable decrease in τ_3_ becomes clearly visible for photoexcitation between 310 and 287 nm (see SI, Figure S5) and the trend continues toward shorter excitation wavelengths, which is discussed further in the next section.

The EAS signal of Path 3 shows a shoulder at around 8.7 eV in the τ_1_ EAS (blue band in Figure 4e) that is addressable to the fast-decaying ^1^π_S4_π_S2_^*^ state. This state converts to the more intense ^1^n_S2_π_S2_^*^ state on an ultrafast time scale. Due to the presence of two ^1^π_S2_π_S2_^*^ and ^1^π_S2_π_S4_^*^ close-lying and overlapping bands, activating Path 4 and Path 2, a minor contribution from the corresponding excited-state minima is recognizable in the EAS spectrum of τ_1_. The τ_1_ EAS shows spectral features in the binding energies associated with three components of the populated ^1^ππ* state minima (Min-^1^π_S4_π_S2_^*^, Min-^1^π_S2_π_S2_^*^, Min-^1^π_S2_π_S4_^*^, vertical blue lines in Figure 4e). The relative structures of τ_2_ and τ_3_ EAS do not change significantly from those observed for Path 4, which supports the interpretation that the decay follows Path 4 for the subsequent ISC dynamics at longer times.

### 2.3 The Fate of the Triplet Manifold

A closer inspection of the three shortest excitation wavelengths in Table 1 shows a significant decrease in the triplet-state lifetime (τ_3_) from the UV-A to the UV-B excitation regions (region 1 and region 2, respectively), posing a question regarding the fate of the triplet state upon UV-B excitation. As discussed earlier, critical differences between Path 1 and Path 4 are the nature of the final occupied triplet state, i.e., ^3^π_S4_π_S4_^*^ versus ^3^π_S2_π_S2_^*^, respectively, and the relaxation coordinate involving in-plane deformation along Path 1 versus sulfur 2 out-of-plane bending along Path 4. Whereas the ^3^π_S4_π_S4_^*^ state is long-lived, depopulation dynamics of the ^3^π_S2_π_S2_^*^ state are observed within the time window of our experiment. According to Path 4 (i) red dashed arrow in Figure 3b, the ISC-^3^π_S2_π_S2_^*^/GS crossing point is situated 0.37 eV above the Min-^3^π_S2_π_S2_^*^ and accumulation of vibrational energy in a sulfur 4 out-of-plane mode during electronic relaxation is a requisite for surmounting this barrier. Therefore, the efficiency of the GS recovery is not only expected to increase with the excitation energy but also show a clear turning point with the onset of Path 3, which launches the dynamics toward Min-^3^π_S2_π_S2_^*^ (by merging with Path 4).

**Figure 5:**
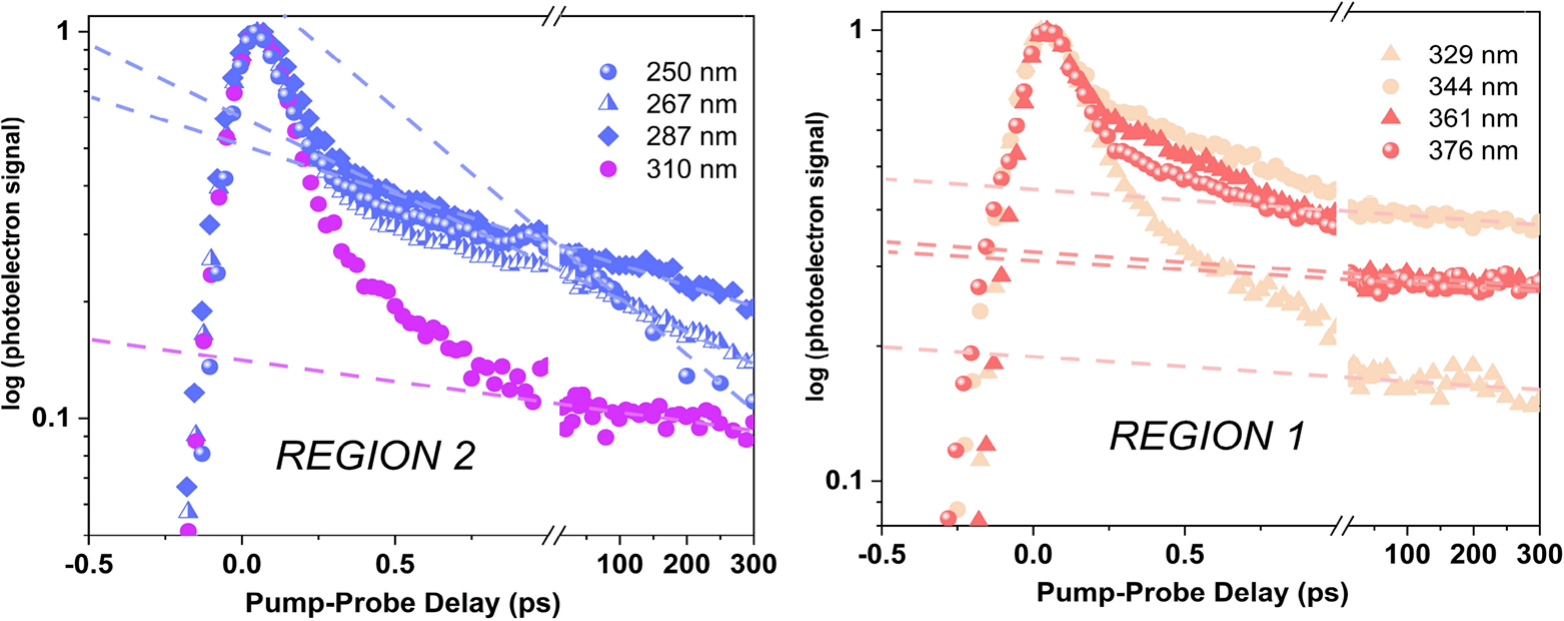
Time traces of all excitation wavelengths for region 1 (right panel) and region 2 (left panel), integrated over the entire spectrum. The total photoelectron signal of each time trace is normalized and plotted on a log scale. For each region, the color scheme from Table 1 and Figure 2 is adopted and indicates the path accessible at the particular excitation wavelength. Circles, triangles, or boxes show the signal, and the colored dashed lines are drawn to indicate the slope of the τ_3_ decay section at long pump–probe delays. In region 1, for excitation wavelengths longer than 320 nm, the slope of the dashed lines shows a negligible wavelength dependence and generally long lifetimes, τ_3_. In region 2, below 320 nm, the steeper slopes toward shorter excitation wavelength can be associated with faster dynamics.

The long-range dynamics observed in the TRPES are investigated to assess the triplet-state deactivation further. For this purpose, the integrated TRPES time traces are plotted in a logarithmic scale in Figure 5 so that the slope of a line through the long-range data visualizes the triplet-state decay time. The limited pump–probe delay range, unfortunately, prevents a more quantitative analysis of the wavelength-dependence of the τ_3_ dynamics (also see Figure S5 in SI). In agreement with the proposed deactivation channels of ^3^π_S2_π_S2_^*^ and ^3^π_S4_π_S4_^*^, two distinct regions are apparent and are shown in two separate panels. In the UV-A excitation region (region 1, right panel), the slopes of the lines are almost unchanged. The associated ^3^π_S4_π_S4_^*^ triplet state survives much longer than two ns, in line with the behavior of singly substituted 4-TU (see Table S3 in the SI). Conversely, a noticeable change in the slope is observed in the UV-B region (region 2). Specifically, the ^3^π_S2_π_S2_^*^ triplet-state lifetime decreases with increasing excitation energy from ns to ps consistent with 2-TU (see Table S3 in the SI).

For the sake of completeness, we further explore the depopulation mechanism of the higher triplet minimum (Min-^3^π_S2_π_S2_^*^) along potential Paths 4(i) and 4(ii). Along Path 4(ii) (red dashed line in Figure 3b), the molecule adiabatically relaxes from the high-energy Min-^3^π_S2_π_S2_^*^ region to the lower-energy Min-^3^π_S4_π_S4_^*^ region on the triplet-state PES. Although this pathway leads to a thermodynamically more stable species by overcoming a small potential barrier (0.21 eV), it requires redistribution of vibrational energy from the hot sulfur 2 bending modes into initially cold sulfur 4 in-plane modes. A similar process has been reported to occur in 2-TU, but for a distinctly different triplet topography with two isoenergetic minima.^57^ The initially populated sulfur 2 out-of-plane minimum resembles Min-^3^π_S2_π_S2_^*^ and barrier crossing to the ring-distorted minimum, comparable to Min-^3^π_S4_π_S4_^*^, which requires a similar vibrational mode change. This process was found to occur on ps to ns timescales. Nonetheless, the study favored continuation along the sulfur 2-out-of-plane coordinate as the primary mechanism leading to the GS repopulation. In analogy, the adiabatic relaxation process to Min-^3^π_S4_π_S4_^*^ in 2,4-DTU, in principle, competes with the triplet depletion via the ISC-^3^π_S2_π_S2_^*^/GS funnel. However, Path 4 (i) is driven by the hot sulfur 2 bending mode and its dominance is more consistent with the significant decrease in the triplet-state lifetime (τ_3_) depicted in Figure 5. Adiabatic decay to the ^3^π_S4_π_S4_^*^ triplet state would imply extended population trapping due to the absence of a low-lying ISC-^3^π_S4_π_S4_^*^/GS crossing point. While TRPES data over an extended pump-probe delay range would be desirable for a more conclusive interpretation of the triplet dynamics, it should be noted that the TRPES technique cannot unambiguously distinguish the photoelectron spectra of the triplet minima, Min-^3^π_S4_π_S4_^*^ and Min-^3^π_S2_π_S2_^*^. According to the values on top of Figure 3b, they exhibit almost identical IPs (6.47 vs 6.51 eV) and intensities (0.79 vs 0.71), respectively. Based on the above discussion, we conclude that GS repopulation in 2,4-DTU favorably proceeds via ISC-^3^π_S2_π_S2_^*^/GS in analogy with the mechanism in 2-TU.

## 3 Conclusions

We combined CASPT2 calculations and femtosecond TRPES experiments of the different photodecay processes induced by illuminating the system at selected wavelengths across the entire UV-A-UV-B LA spectrum. A clear picture of the reasons behind the non-negligible wavelength-dependent changes in the IC and ISC events is gained. The TRPES technique can track both bright and dark states in the relaxation dynamics. This is a crucial advantage for investigating the excited-state dynamics of doubly-thionated bases, which exhibit four optically bright ^1^ππ* and four dark ^1^*n*π* states in the UV window.

**Scheme 1:**
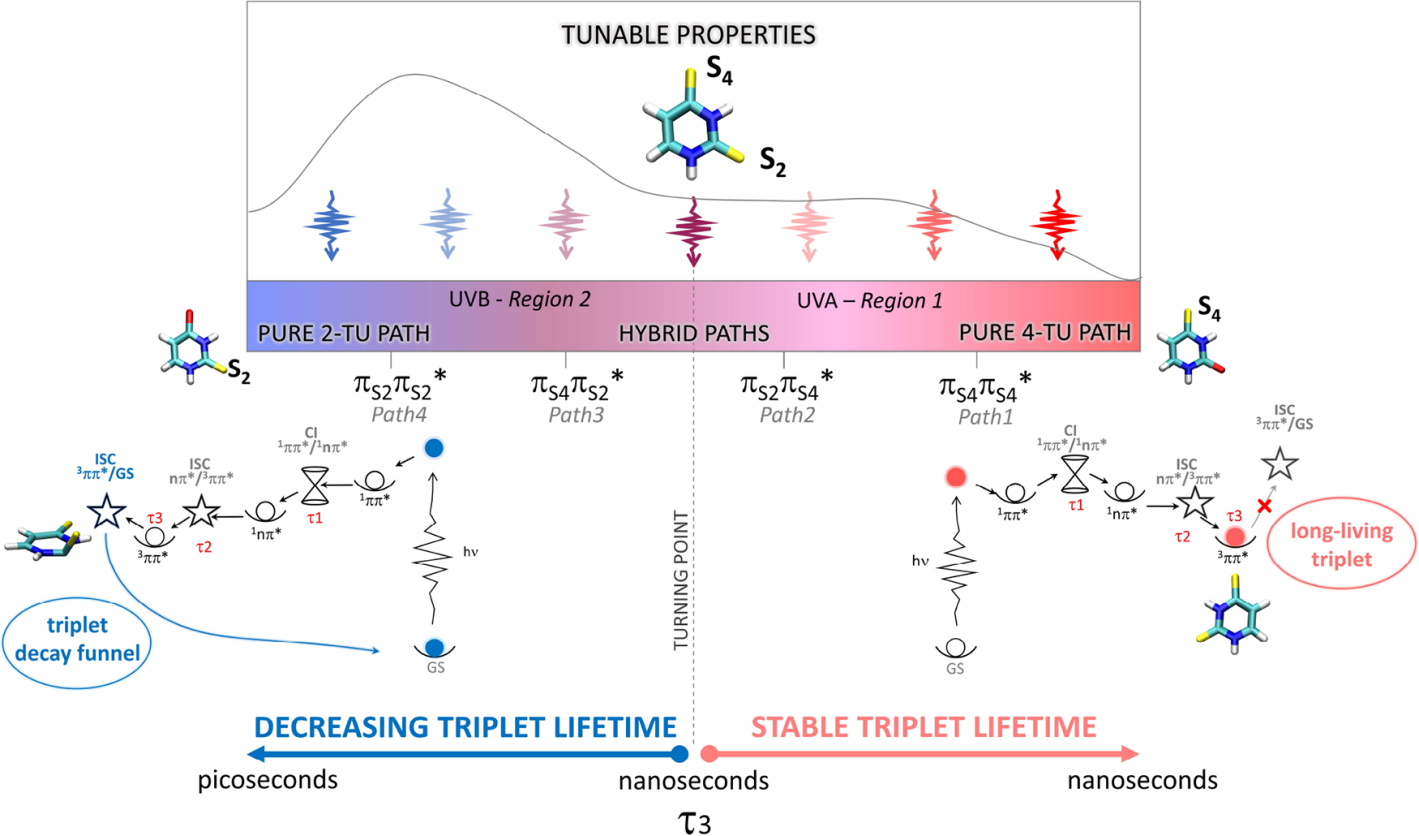
An overview of the tunable properties of the double thionated 2,4-DTU. The photoinduced properties are smoothly changing by moving across the LA spectrum. The absorption band’s extremes closely resemble the singly substituted 4-TU and 2-TU behaviors

The photodynamics can be categorized as follows:

a. The detailed CASPT2 calculations describe four decay paths (Path 1, Path 2, Path 3, and Path 4), which originate from four bright ^1^ππ* excited states (^1^π_S4_π_S4_^*^,^1^π_S2_π_S4_^*^,^1^π_S4_π_S2_^*^, and ^1^π_S2_π_S2_^*^) across the UV-A to UV-B excitation region. Combined with discontinuous trends observed in the experimental time constants, this allowed us to group the wavelength-dependent TRPES by their common decay paths. The color code highlights this categorization in Figure 2. Typically, in the presence of a barrier along a relaxation path, a decrease in time constants is observed for higher excitation energies. Moreover, for ISC processes, the molecular out-of-plane vibrational modes can potentially affect the SOC between the singlet and triplet states, systematically increasing the ISC time rates at decreasing pump wavelengths.^53^ Therefore, the observed discontinuities suggest the division into sub-groups associated with alternative decay paths based on a change in the nature of the participating electronic states.
b. Upon photoexcitation at the two extremes of the LA spectrum (at λ > 360 nm or λ < 287 nm), the system populates the so-called “pure” decay mechanisms (Path 1 and Path 4), which resemble those of the singly substituted 4-TU and 2-TU (Table S3 in the SI). While the former is characterized by a slow ^1^*n*_S4_π_S4_^*^/^3^π_S4_π_S4_^*^ ISC and a population trapping in the ^3^π_S4_π_S4_^*^ triplet minimum, the latter is characterized by a fast ^1^*n*_S2_π_S2_^*^/^3^π_S2_π_S2_^*^ ISC and a short-lived ^3^π_S2_π_S2_^*^ triplet state along with sulfur 2 out-of-plane bending that opens a gateway for GS repopulation on a few hundreds of ps timescale (see Scheme 1).
c. The relaxation mechanisms at intermediate wavelengths of the LA spectrum (within 310 nm < λ < 344 nm) show strong non-adiabatic coupling between electronic states with mixed orbital transitions of different sulfurs (Path 2 and Path 3). However, at longer times, they eventually populate either the Min-^3^π_S4_π_S4_^*^ or Min-^3^π_S2_π_S2_^*^ triplet minima, similar to the “pure” pathways. Consequently, the multiple relaxation mechanisms excited simultaneously at wavelengths 310 nm < λ < 344 nm lead to a complex trend in the TRPES time constants (Table 1 and Figure 2).
d. A decay of the triplet-state (^3^π_S2_π_S2_^*^) signal is observed at high excitation energies, starting from λ lower than ∼310 nm, as documented in Figure 5. This is addressed to Path 3 and Path 4 in Figure 3b. The fast depopulation of this triplet state can be justified by a high probability of accessing the ISC region (ISC-^3^π_S2_π_S2_^*^/GS), which directly funnels the population to the GS and is characterized by a sulfur 2 out-of-plane deformation (Path 4 (i)). An increase in excitation energies corresponds to a reduction of the ^3^π_S2_π_S2_^*^ lifetime, as the excess vibrational energy in the molecule and sulfur 2 modes helps surmount the barrier to the crossing point. An alternative adiabatic decay route to the lowest triplet-state minimum Min-^3^π_S4_π_S4_^*^ (Path 4 (ii)) was excluded due to the absence of a long-lived TRPES signal.

The overall wavelength-dependent photodynamics are visualized in Scheme 1. Based on the above-itemized differences arising from the excitation energy, we observed a clear qualitative separation of the 2,4-DTU LA spectrum into region 1 and region 2 that matches the singly substituted 4-TU and 2-TU absorption bands, respectively (see Figure 1). In the UV-A (region 1) excitation, the behavior of the molecule is more similar to 4-TU: the initial ^1^*n*π* → ^1^ππ* CI barrier/entropic trap (Path 1 and Path 2 in Figure 3a) leads to a slower ISC process (τ_2_) followed by a longer-lived ^3^π_S4_π_S4_^*^ final triplet state (τ_3_) because of the absence of triplet → GS ISC regions at accessible energies. Conversely, the UV-B (region 2) excitation, which leads to a faster ISC time and shorter Min-^3^π_S2_π_S2_^*^ triplet lifetime, clearly resembling the 2-TU behavior (Figure 3b). In this case, the shorter triplet lifetime is due to a low-lying ISC region along the sulfur 2 bending mode, as previously documented for the 2-TU derivative. As a result, by selective excitation of the doubly substituted uracil (2,4-DTU) in region 1 or region 2, one can obtain tunable photodynamics closely resembling the singly substituted 4-TU and 2-TU behaviors, as summarized in Scheme 1. The turning point of the decay mechanism is located between 329 and 287 nm (∼310 nm), corresponding to the gray vertical dashed line in Figure 1.

From an application point of view, 2,4-DTU can supply significant advantages compared to singly substituted Uracils. Most importantly, different photoinduced responses can be obtained by a minor change in excitation wavelength across the UV-A and UV-B windows. Lifetimes and formation rates of triplet states become tunable properties that can easily be controlled to match the criteria necessary for the desired biological or technological application. The transferability of the mechanistic details from the gas phase to a wide range of environments is confirmed and discussed in the SI (see Section S7). 2,4-Dithiothymine (2,4-DTT) is expected to display similar wavelength-tunable photoproperties given the close resemblance of the absorption spectra,^50,58^ electronic structures and excited-state potentials of thiothymines and thiouracils.^19,41,43,58^ In fact, prospective photo-chemotherapeutic applications of 2,4-DTT, which rely on the highly efficient ISC dynamics and long-lived triplet state, have been demonstrated experimentally.^10^

## 4 Experimental and Theoretical Details

### 4.1 Time-Resolved Photoelectron Spectroscopy Setup

More details on the TRPES experiment can be found in previous studies.^8,59,60^ The experiment includes a femtosecond laser system tunable in the UV and a magnetic bottle for gas-phase photoelectron spectroscopy. The laser system consists of a Coherent Inc. Mira-seeded Legend Elite HE amplifier with an output of 130 fs, 3.1 mJ/pulse, and a 1 kHz repetition rate centered at a wavelength of 802 nm. A Traveling-wave Optical Parametric Amplifier (TOPAS-C) is tuned to pump wavelengths of 329, 310, 287, and 250 nm. The amplifier fundamental’s third and second harmonic served as a 267 nm pump and a 401 nm probe, respectively. Since one-photon excitation but two-photon ionization were desired, i.e., a 1 + 2′ process, pump pulse energies were limited to 1–2.5 μJ/pulse, and the probe was set to 15 μJ/pulse, except for the 267 nm scan, which was 6 μJ/pulse. 2,4-DTU solid powder from Sigma-Aldrich (98%) was placed inside a quartz sample holder and heated to 175 ° C. The molecules were then continuously expanded with a helium carrier gas under non-clustering conditions. After passing through two skimmers and an intermediate differential pumping stage, the molecules were transferred into the spectrometer region, where the molecular beam and two focused laser beams overlapped. Photoelectron spectra were derived from electron time-of-flight measurements and energy calibrations based on 1,3-butadiene (BD) with known cationic vibrational energy levels to determine photoelectron kinetic energies (*E*_kin_).^61^ BD scans also served for timing calibrations, determining the temporal overlap of pump and probe pulses (time-zero) and a Gaussian instrument response function (IRF) of around 180 fs FWHM. Photoelectron spectra are plotted as a function of the electron BE (eV) instead of electron kinetic energy to facilitate the direct comparison of the spectra to calculated IPs. The electron BE is calculated by subtracting the electron kinetic energy from the total photon energy in the following equation in a 1 + 2′ multiphoton process.
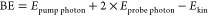
1where BE is the electron binding energy, *E*_kin_ is the measured photoelectron kinetic energy, *E*_pump photon_, and *E*_probe photon_ are the photon energies for pump and probe wavelengths, respectively.

The 376, 361, and 344 nm TRPES data reproduced from earlier work was obtained using the same experimental set up and recorded under similar experimental conditions.^8^

### 4.2 Computational Details

The ground state (GS) minimum geometry was obtained with the Møller–Plesset second-order perturbation theory (MP2). CASPT2 multireference dynamically correlated methods systematically modeled the different excited-state decay pathways and the corresponding TRPES spectra based on the minimum energy paths involving all the characterized CPs, CIs, and ISCs involved in the deactivation induced at the different excitation wavelengths. The excited-state geometry optimizations have been performed with numerical gradients at the CASPT2 level, as implemented in OpenMolcas^62,63^ using the optimizer of Gaussian 16^64^ through its interface with COBRAMM.^65−67^ The active space used in geometry optimization includes the sulfur lone pairs and all the valence π-orbitals resulting in a total of 14 electrons in 10 orbitals (see Figure S2 in the SI). The employed ANO basis set adopts the following contractions: 5s4p2d1f for sulfur, 4s3p2d1f for carbon/oxygen/nitrogen, and 3s2p1d for hydrogen atoms.^68^ CI optimizations were performed with the gradient projection algorithm by Bearpark et al.,^69,70^ implemented in COBRAMM. The required non-adiabatic vectors have been determined numerically from wavefunction overlaps through the RASSI routine of OpenMolcas. Thereby, we used perturbatively modified CASSCF wavefunctions obtained in the MS-CASPT2 routine.

All optimized CPs, CIs, and ISCs energies were refined with MS-CASPT2 vertical calculations,^71^ including a larger active space (18 electrons, 14 orbitals), extended by two bonding and two antibonding s-orbitals on the sulfurs (see Figure S2). Together with an enlarged ANO-RCC basis set, using 6s5p3d2f1g contraction on sulfur, 5s4p3d2f1g on carbon/oxygen/nitrogen, and 4s3p2d1f on hydrogen atoms. State-averaging (SA) over nine states have obtained the final vertical energies. The ionization potential electron affinity (IPEA) shift^72,73^ was set to 0.0, the imaginary shift to 0.2 a.u.^74^ Transition dipole moments (TDMs) have been calculated through the RASSI routine using the perturbatively modified CASSCF wavefunctions obtained in the MS-CASPT2 routine.^75^ The MS-CASPT2 level of theory was chosen based on a preliminary comparison with XMS-CASPT2, documented in Figure S1.

To do the ionization energy calculations for the TRPES spectra for each singlet and triplet CP, nine ionic doublet states were calculated at the MS-CASPT2(17,14)/ANO-RCC level with the aim of capturing multiple ionization channels.^71^ Dyson norms were computed with OpenMolcas for all singlet–doublet and triplet–doublet state pairs to evaluate and simulate the intensities of the photoelectron spectra.

The electron BE at each CP, CI, and ISC was determined as:
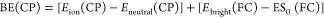
2where [*E*_ion_(CP) – *E*_neutral_(CP)] corresponds to the energy difference between a particular populated neutral CP and the equivalent target ionic state, giving the IPs documented in correspondence with all CPs or crossing regions (CIs or ISCs) on top of the four decay Paths of Figure 3. The term [*E*_bright_(FC) – ES_0_ (FC)] represents the initial excitation energy in the Franck–Condon (FC) region. The calculated BE is, therefore, equivalent to the BE definition in the experimental spectra:
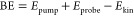
3*E*_kin_ is the measured photoelectron kinetic energy, and *E*_pump_ and *E*_probe_ are the photon energies in the one-photon pump and two-photon probe processes. Therefore, the energies of experimental and simulated photoelectron bands are directly comparable and their intensities reflect the excited-state populations at CP along the relaxation paths.

## Supporting Information Available

Absorption spectrum and vertical energies for evaluation of level of theory; additional theoretical details: active space orbitals, detailed decay paths, water solvent effects, and Cartesian coordinates; additional experimental data and analysis: time-resolved photoelectron spectra, exponential decay model and fit function, time traces, and associated spectra; and additional plots to support the interpretation (PDF)
