# Supplementary material for: Double Thionated Pyrimidine Nucleobases: Molecular Tools with Tunable Photoproperties
Source: J Am Chem Soc. 2023 May 25;145(22):11945–58. doi: 10.1021/jacs.2c12061 (PMC10251510; doi:10.1021/jacs.2c12061)
Supplement: Supplementary file 1 — ja2c12061_si_001.pdf [file ja2c12061_si_001.pdf]

Double Thionated Pyrimidine Nucleobases: Molecular Tools with Tunable Photoproperties

Abed Mohamadzade[a#], Artur Nenov[b#], Marco Garavelli[b], Irene Conti[b*], Susanne Ullrich[a*]
[a]Department of Physics and Astronomy, University of Georgia, Athens, GA 30605, USA.
[b]Dipartimento di Chimica Industriale "Toso Montanari", Università di Bologna, 40136 Bologna, Italy.
#contributed equally
*corresponding authors
E-mail: irene.conti@unibo.it E-mail: ullrich@uga.edu

SUPPORTING INFORMATION

Contents

S1. 2,4-DTU Linear Absorption Spectrum ... 2
S2. CASPT2 Active Space Orbitals. ... 3
S3. 2D Photoelectron Signals ... 4
S4. TRPES Fit Results ... 5
4.1 Fit Results Using a Two-Step Sequential Exponential Decay Model ... 5
4.2 Fit Function ... 6
4.3 Time Traces and Associated Spectra ... 7
S5. TRPES Spectra Interpretation ... 9
5.1 Computed S_0-Photoelectron Spectra for 2,4-DTU ... 9
5.2 Binding Energy Estimation for Assignment of EAS ... 10
S6. Detailed Decay Paths ... 14
S7. Water Solvent Effect on Triplet Minima and Intersystem Crossings ... 15
S8. Comparison of 2-TU, 4-TU and 2,4-DTU Decay Constants ... 16
S9. Cartesian Coordinates ... 17
S10. References ... 24

S1. 2,4-DTU Linear Absorption Spectrum

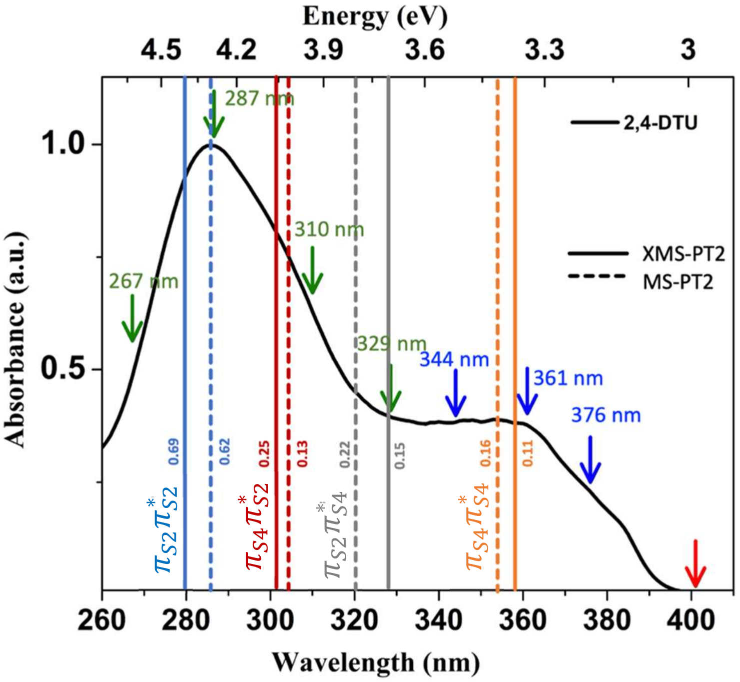

Figure S1. The absorption spectrum of 2,4-DTU was recorded in the CCl_4 solution. Calculated XMS-PT2 and MS-PT2 energy values show a direct comparison between two levels of theory to validate and choose the best method to adopt.

	MS-PT2 energy values (eV)		XMS-PT2 values	
State	singlet	triplet	singlet	triplet
GS	0.000		0.000	
$\pi_{S4}\pi^*_{S4}$ (bright)	3.511	2.522	3.467	2.556
$\pi_{S2}\pi^*_{S4}$ (bright)	3.856	3.352	3.786	3.471
$\pi_{S4}\pi^*_{S2}$ (bright)	4.072	3.041	4.105	3.094
$\pi_{S2}\pi^*_{S2}$ (bright)	4.318	3.744	4.424	3.769
$n_{S4}\pi^*_{S4}$ (dark)	2.726	2.653	2.614	2.551
$n_{S2}\pi^*_{S4}$ (dark)	4.149	4.132	3.894	3.872
$n_{S4}\pi^*_{S2}$ (dark)	3.548	3.489	3.493	3.415
$n_{S2}\pi^*_{S2}$ (dark)	4.111	4.113	3.937	3.910

Table S1. Vertical excitation in the minimum ground state geometry (Franck-Condon) calculated at the MS-CASPT2 and XMS-CASPT2 levels. The active space includes the sulfur lone pairs and all the valence p-orbitals, extended by two bonding and two antibonding S orbitals on sulfurs resulting in a total of 18 electrons in 14 orbitals. ANO-RCC basis set, showing 6s5p3d2f1g contraction on sulfur, 5s4p3d2f1g on carbon/oxygen/nitrogen, and 4s3p2d1f on hydrogen atoms. Vertical energies have been obtained by state-averaging on 9 states.

S2. CASPT2 Active Space Orbitals.

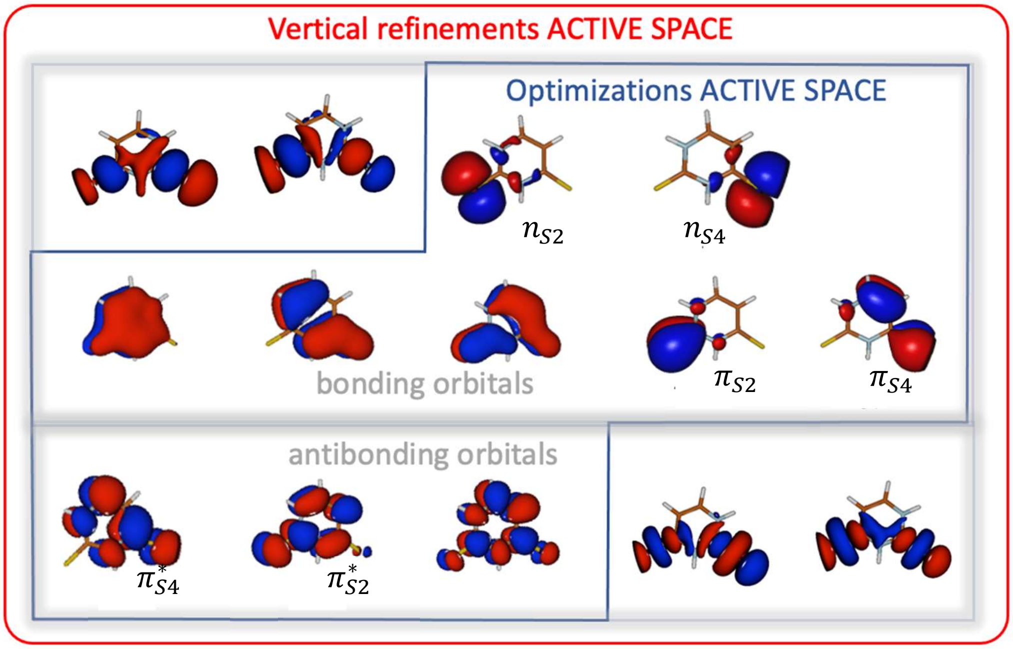

Figure S2. Active space adopted for the CASPT2 optimizations (inside the blue line) and in the energy refinement of each characterized critical point or conical intersection (inside the red line).

S3. 2D Photoelectron Signals

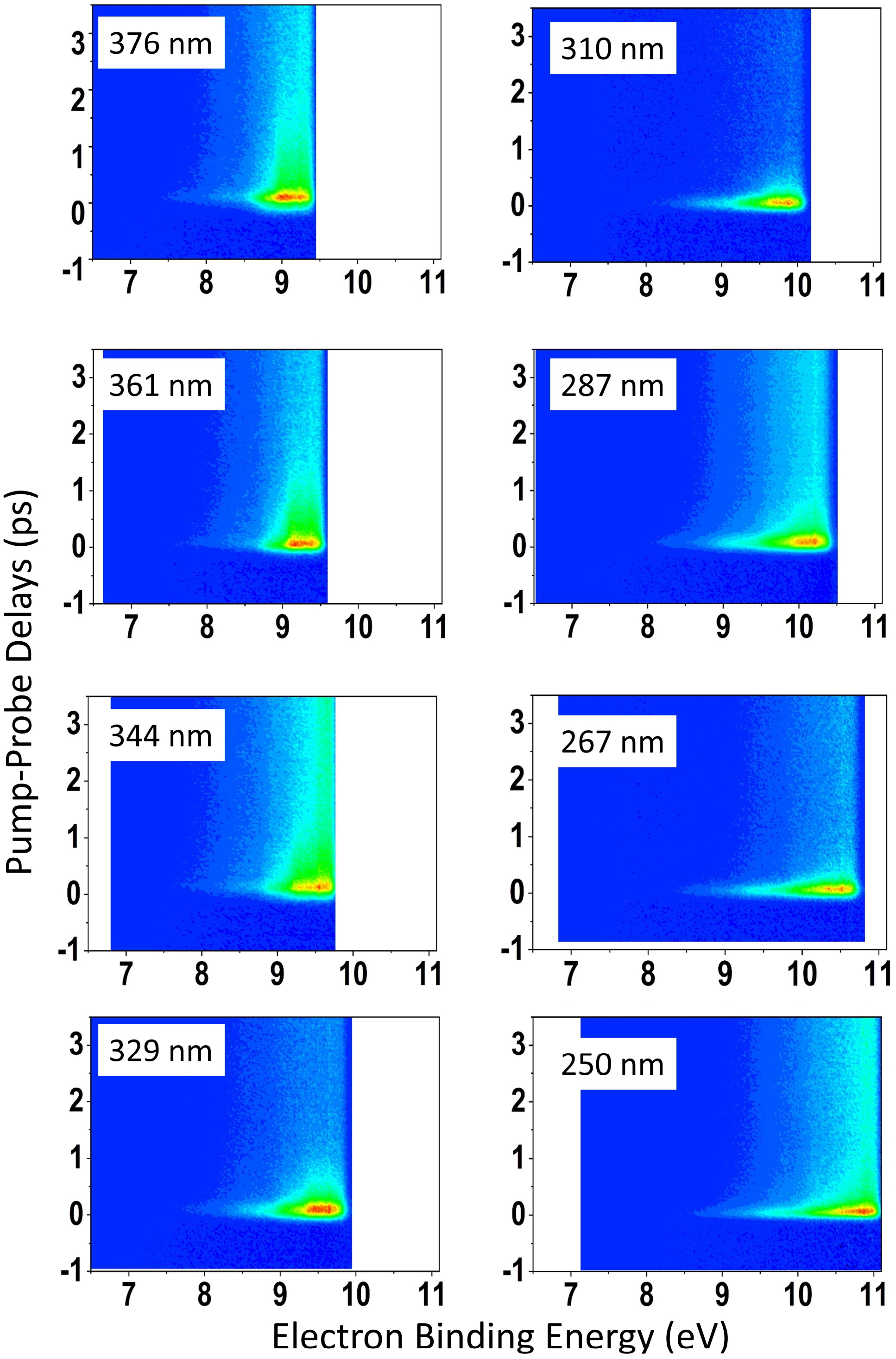

Figure S3. 2D maps of the TRPES signal. The experimental data are recorded in the range of -1 ps to 300 ps but are truncated to the first 3.5 ps of pump-probe delays for better visibility of the region responsible for the ultrafast dynamics clearer. The maps are plotted as a function of electron binding energy (BE, in eV) on the x-axis and pump-probe delays (ps) on the y-axis. The color scheme for the 2D maps is chosen to show blue for zero intensity and red for the maximum signal intensity, and the color level is set to the same value for all the 2D plots.

S4. TRPES Fit Results

4.1 Fit Results Using a Two-Step Sequential Exponential Decay Model

Figure S4 provides justification for using a three-step sequential exponential decay model. The graphs show two-step sequential fits on the time traces of the integrated TRPES over the extended pump-probe delay range. Fit results with only two exponentials inadequately describe the dynamics with apparent deviations either in the region around zero pump-probe delay or intermediate pump-probe delay region (0.5 ps to 1 ps).

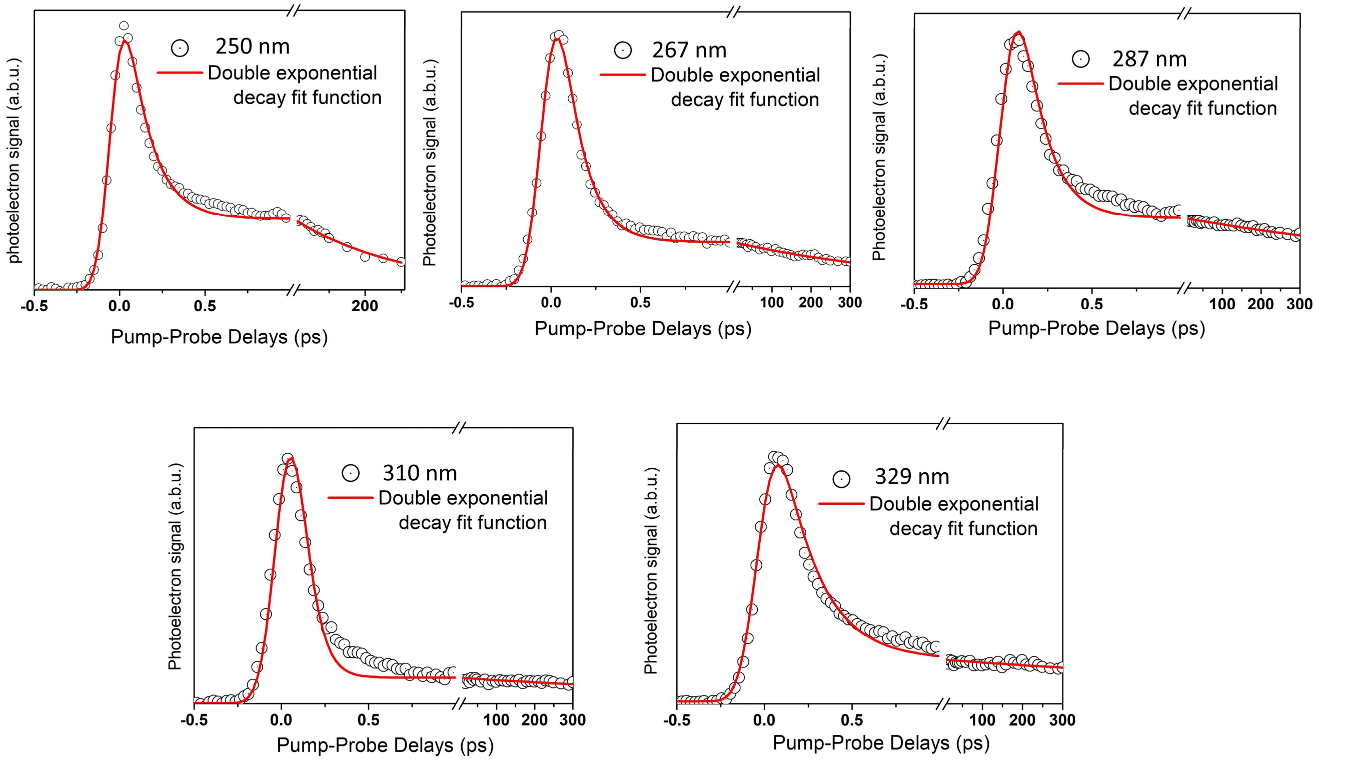

Figure S4. Fits on integrated TRPES data over the entire pump-probe delay range using a sequential decay function with two exponentials. Integrated TRPES signal (black circles) and total fit (red line) are shown for five pump wavelengths employed in experiments in this study. The same justification was shown for the three wavelengths from our previous publication. [1]

Based on the calculated minimum energy pathways, and experimental results, the 310 nm is introduced as a turning point for the excited state dynamics of 2,4-DTU in terms of the final occupied triplet state, which leads to a shortened triplet state lifetime. Figure S5 below plots the time traces of 287, 310, and 329 nm scans on a lin-log scale, and a linear fit on the long-range dynamics emphasize the change in the slope going from 329 nm to 287 nm. The time constant of the triplet state lifetime can be extracted as an inverse of the calculated slope, which leads to 1, 1.6, and 2.2 ns for 287, 310, and 329 nm, respectively.

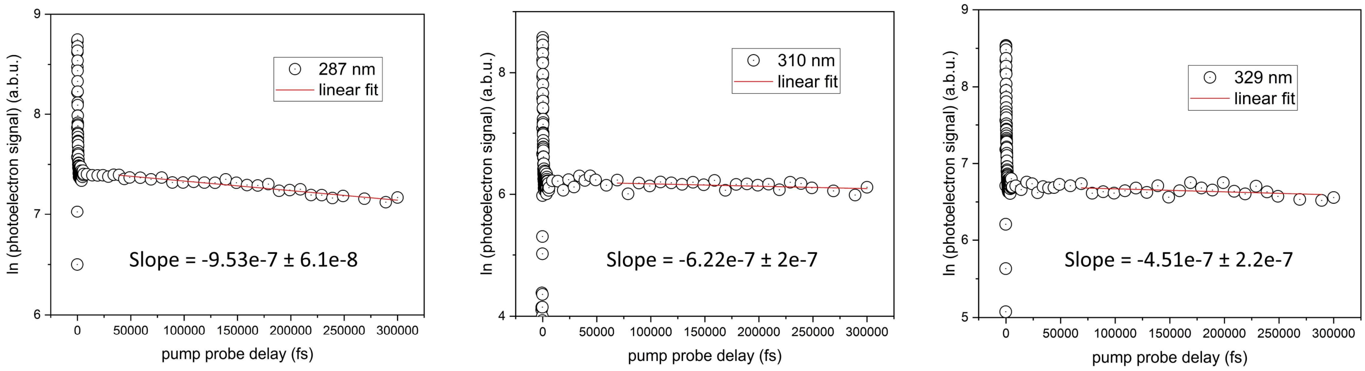

Figure S5. Integrated time traces of 287, 310, and 329 nm scans are plotted on a lin-log scale. The slope of a linear fit on the long-range dynamic is also shown as an inset.

4.2 Fit Function

The three-step sequential exponential decay fit function was used to analyze the TRPES integrated time traces, shown below. Where $k_1 = \frac{1}{\tau_1}$, $k_2 = \frac{1}{\tau_2}$, and $k_3 = \frac{1}{\tau_3}$ are the rate constants for the process A→B→C→D and $sA(E)$,$sB(E)$, and $sC(E)$ represent decay-associated photoelectron spectra. The instrument response function IRF is described by the FWHM of the Gaussian cross-correlation.

$$
\begin{aligned}
x(t,E) = \; & sA(E) * \left(e^{\left(\frac{IRF*k_1}{4\sqrt{ln2}}\right)^2} * e^{-k_1 t} * \frac{1}{2}\left(1 + erf\left(\frac{2\sqrt{ln2}}{IRF}t - \frac{IRF*k_1}{4\sqrt{ln2}}\right)\right)\right) + sB(E) \\
& * \left(e^{\left(\frac{IRF*k_1}{4\sqrt{ln2}}\right)^2} * \frac{k_1}{k_2 - k_1} e^{-k_1 t} * \frac{1}{2}\left(1 + erf\left(\frac{2\sqrt{ln2}}{IRF}t - \frac{IRF*k_1}{4\sqrt{ln2}}\right)\right) + e^{\left(\frac{IRF*k_2}{4\sqrt{ln2}}\right)^2} * \frac{k_1}{k_1 - k_2} e^{-k_2 t} \right. \\
& \left. * \frac{1}{2}\left(1 + erf\left(\frac{2\sqrt{ln2}}{IRF}t - \frac{IRF*k_2}{4\sqrt{ln2}}\right)\right)\right) + sC(E) \\
& * \left(- e^{\left(\frac{IRF*k_1}{4\sqrt{ln2}}\right)^2} * \frac{k_3 k_2}{k_1^2 + (k_2 - k_1)k_3 - k_1 k_2} e^{-k_1 t} * \frac{1}{2}\left(1 + erf\left(\frac{2\sqrt{ln2}}{IRF}t - \frac{IRF*k_1}{4\sqrt{ln2}}\right)\right) + e^{\left(\frac{IRF*k_2}{4\sqrt{ln}}\right)^2} \right. \\
& * \frac{k_1 k_3}{k_2^2 - (k_2 - k_1)k_3 - k_1 k_2} e^{-k_2 t} * \frac{1}{2}\left(1 + erf\left(\frac{2\sqrt{ln2}}{IRF}t - \frac{IRF*k_2}{4\sqrt{ln2}}\right)\right) + e^{\left(\frac{IRF*k_3}{4\sqrt{ln2}}\right)^2} \\
& \left. * \frac{k_1 k_2}{k_3^2 - (k_1 + k_2)k_3 + k_1 k_2} e^{-k_3 t} * \frac{1}{2}\left(1 + erf\left(\frac{2\sqrt{ln2}}{IRF}t - \frac{IRF*k_3}{4\sqrt{ln2}}\right)\right)\right)
\end{aligned}
$$

4.3 Time Traces and Associated Spectra

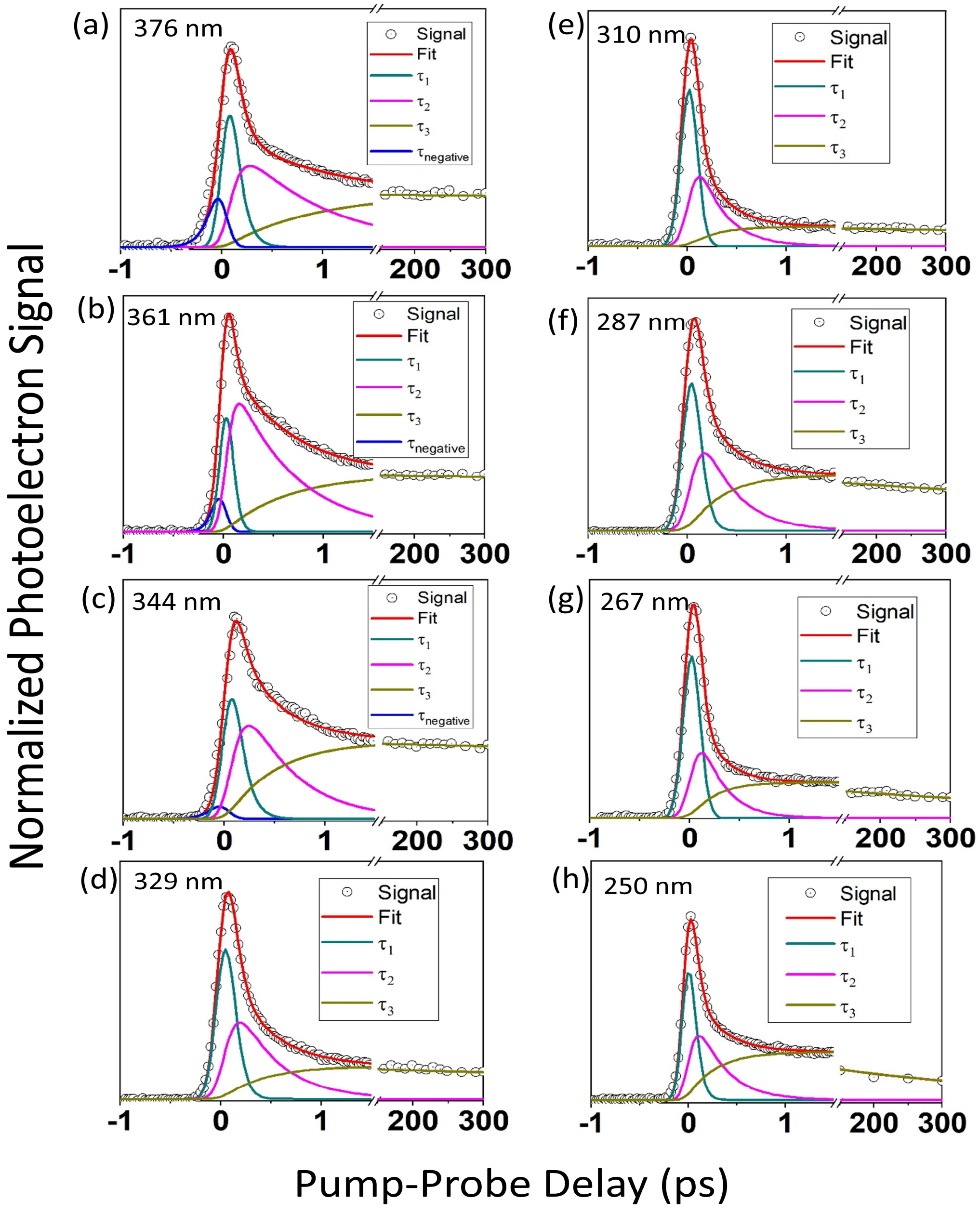

Figure S6. Experimental kinematics TRPES fit results are presented in the order of decreasing excitation wavelength from the top to the bottom. Black circles show the signal, the red line is the total fit, and blue, purple, and dark yellow lines are contributing time constants. The dark blue component in the first three wavelengths is the probe-pump signal due to two-photon excitation with probe.

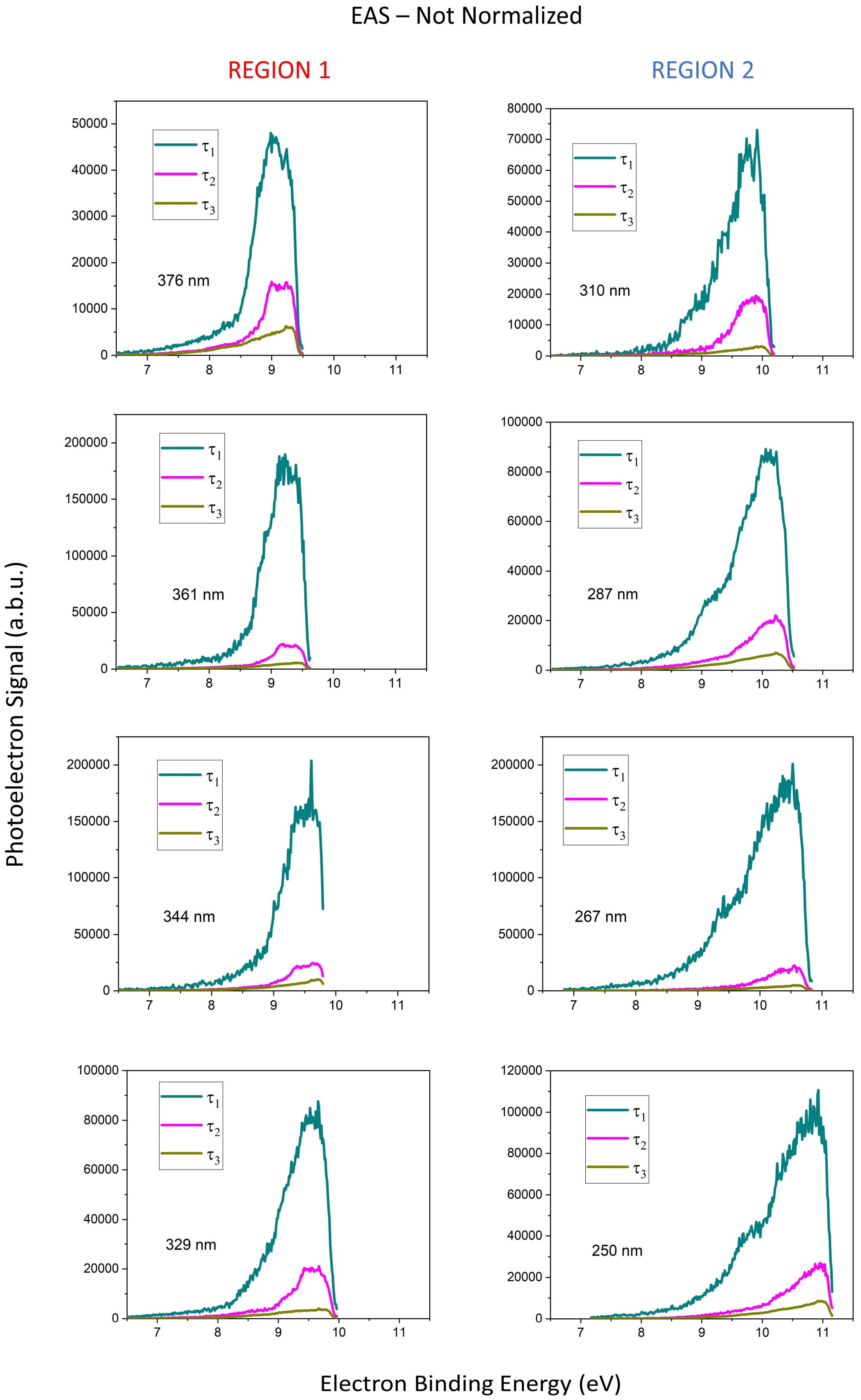

Figure S7. Not-normalized Evolution-Associated Spectra (EAS), extracted from the global lifetime analysis of the experimental TRPES (Figure S3, ESI). More details are provided in the discussion of Figure 4 in the main text.

S5. TRPES Spectra Interpretation

The change in binding energy (BE) along the relaxation path is characterized by vertical CASPT2 MS-CASPT2/ANO-RCC calculations, using active space (17,14) and (18,14), on the ionic and neutral forms, respectively. BE corresponds to the energy difference between a particular populated neutral critical point and the corresponding target ionic states, added to the initial excitation energy in the Franck-Condon (FC) region. Photoelectron signals are expected at the electron binding energies indicated as solid lines in Figure 4 in the manuscript, calculated based on the different adopted excitation energies. This information provides the basis for identifying the electronically excited states participating in the three-step relaxation process. The Dyson amplitude calculations approximate photoelectron intensities between states that differ by exactly one in their number of electrons. These values are indicated in parenthesis, close to the BE vertical line, in Figure 4 (main text). The ionization correlations between electronically excited and ionic states have been considered. The dominant contributions to the photoelectron spectra from the GS minimum are characterized by an n-hole on the sulfur atom for D_0 (lowest ionic state) and a π-hole for D_1 (second ionization channel) in the singly thionated systems.[2] Considering the orbital characteristics of 2,4-DTU with contribution from both sulfurs, in general, two $n\pi^*$ and two $\pi\pi^*$ states are expected to ionize into the four $D_{0\text{-}3}$ states, characterized by n and π holes on Sulfur 2 and 4, respectively.

5.1 Computed S_0-Photoelectron Spectra for 2,4-DTU

The first 12 ionization channels (D_0-D_{11}) of the 2,4-DTU were calculated by L. Gonzalez et al.[2] and show predominant ionization from orbitals on sulfur and the pyrimidine ring, respectively labeled as "S" and "Pyr" (Figure S8). In the following picture, we validate our method, showing that we reproduce the D_0 band ($^1\pi_{S4}\pi^*_{S4}$) at the CASPT2 level without applying any energy shift.

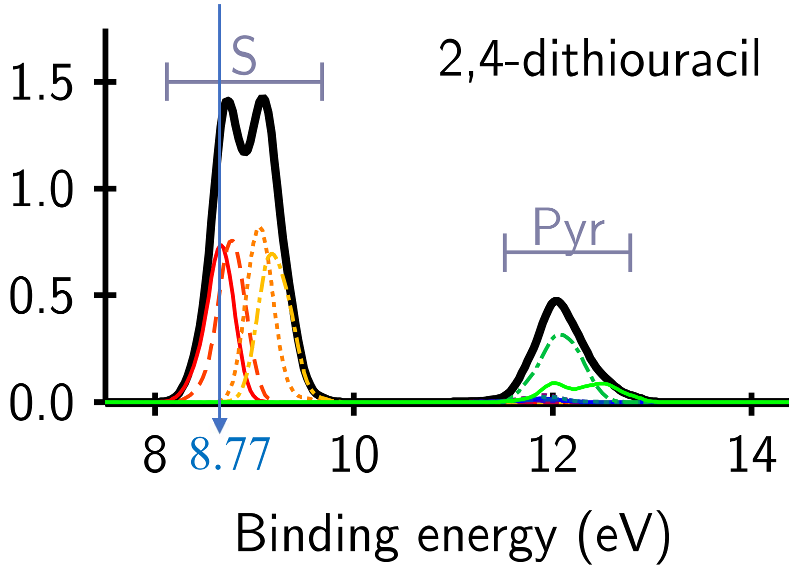

Figure S8: The simulated ground state photoelectron spectrum of 2,4-DTU. Reprinted from Ruckenbauer, M.; Mai, S.; Marquetand, P.; González, L. Photoelectron spectra of 2-thiouracil, 4-thiouracil, and 2, 4-dithiouracil. *The Journal of Chemical Physics* **2016**, *144* (7), 074303, with the permission of AIP Publishing. The colored solid and dashed lines contributing to the signal peaks are calculated ionization channels. The solid red line is the D_0 with an n orbital character, and the close-lying red dashed line is the second ionization channel D_1 with π orbital character. The vertical blue arrow indicates the CASPT2 calculated binding energy (BE) we obtained from the lowest $^1\pi_{S4}\pi_{S4}{}^*$ state in the Franck-Condon region, pumping at 361 nm excitation energy. Our $^1\pi_{S4}\pi_{S4}{}^*$ calculated BE matches exactly with the two lowest components of the first band of the calculated ground state photoelectron spectrum for 2,4-DTU[2]: This double-peak band in the range of 8-10 eV derives from the orbitals localized on sulfur; the other band is centered at about 12 eV, due to ionization from pyrimidine ring orbitals. Also, the experimental photoelectron spectra in Figure S3 concur with the calculated sulfur-addressed signals in terms of the energy range. This is a good confirmation that our calculations reproduce the photoelectron spectra in a proper way.

5.2 Binding Energy Estimation for Assignment of EAS

Binding energies and Dyson norms have been evaluated on singlet, triplet and ionic states calculated at MS-CASPT2(18,14)/ANO-RCC level (MS-CASPT2(17,14)/ANO-RCC for ionic states), averaging on the first 9 states. This allows the evaluation of transitions to a large number of ionic states, which are characterized by holes in different binding orbitals. The following Table S2 only reports binding energies associated with non-negligible intensity values for each critical point (Dyson norms > 0.1). The same values are also included in Figure 4. Usually, the most intense transition for a particular excited state corresponds to an ionic state where the electron in the antibonding orbital is removed, leaving a hole in the π or n binding orbital.

Table S2. MS-CASPT2-calculated energies at the excited state minima, ionization energy, and vibrational energy can be used to estimate the electron binding energy signal in the photoelectron spectra for each geometry. The ionization energy is calculated as the required energy to remove an electron from the excited state minimum. The vibrational energy is calculated based on the subtraction of the excitation energy and minimum excited state energy. For each wavelength, only the relevant geometries involved in the corresponding relaxation *Paths* of Figure 3 are shown. If two ionization energies (belonging to the BE spectral window) with non-negligible intensity values are addressed to one geometry, two close ionization bands exist for the same critical point.

Geometry	Excited state energy (eV)	Vibrational excitation (eV)	Ionization energy (eV)	Binding energy (eV) = Ionization energy + Excited state energy + Vibrational energy
376 nm (3.3 eV)				
Min-$^1\pi_{S4}\pi^*_{S4}$	3.23	0.07	5.66	8.96
Min-$^1n_{S4}\pi^*_{S4}$	2.49	0.81	6.12	9.42
Min-$^3\pi_{S4}\pi^*_{S4}$	2.27	1.03	6.47	9.77
361 nm (3.4 eV)				
Min-$^1\pi_{S4}\pi^*_{S4}$	3.23	0.17	5.66	9.06
Min-$^1n_{S4}\pi^*_{S4}$	2.45	0.95	6.12	9.52
Min-$^3\pi_{S4}\pi^*_{S4}$	2.27	1.13	6.47	9.87
344 nm (3.6 eV)				
Min-$^1\pi_{S2}\pi^*_{S4}$	3.23	0.37	5.55	9.15
Min-$^1\pi_{S4}\pi^*_{S4}$	3.23	0.37	5.66	9.26
Min-$^1n_{S4}\pi^*_{S4}$	2.45	1.15	6.12	9.72
Min-$^3\pi_{S4}\pi^*_{S4}$	2.27	1.33	6.47	10.07
329 nm (3.8 eV)				
Min-$^1\pi_{S2}\pi^*_{S4}$	3.23	0.57	5.55	9.35
Min-$^1\pi_{S4}\pi^*_{S4}$	3.23	0.57	5.66	9.46
Min-$^1n_{S4}\pi^*_{S4}$	2.45	1.35	6.12	9.92
Min-$^3\pi_{S4}\pi^*_{S4}$	2.27	1.53	6.47	10.27
310 nm (4.0 eV)				
Min-$^1\pi_{S4}\pi^*_{S2}$	3.92	0.08	4.81	8.81
Min-$^1\pi_{S2}\pi^*_{S4}$	3.23	0.77	5.55	9.55
Min-$^1\pi_{S2}\pi^*_{S2}$	3.65	0.35	5.90	9.9
Min-$^1n_{S4}\pi^*_{S4}$	2.45	1.55	6.12	10.12
Min-$^1n_{S2}\pi^*_{S2}$	3.17	0.83	6.23	10.23
			6.34	10.34
Min-$^3\pi_{S4}\pi^*_{S4}$	2.27	1.73	6.47	10.47
Min-$^3\pi_{S2}\pi^*_{S2}$	2.85	1.15	6.5	10.51
287 nm (4.3 eV)				
Min-$^1\pi_{S4}\pi^*_{S2}$	3.92	0.38	4.81	9.11
Min-$^1\pi_{S2}\pi^*_{S2}$	3.65	0.65	5.90	10.2
Min-$^1n_{S2}\pi^*_{S2}$	3.17	1.13	6.23	10.53
			6.34	10.64
Min-$^3\pi_{S2}\pi^*_{S2}$	2.85	1.45	6.51	10.81
267 nm (4.6 eV)				
Min-$^1\pi_{S4}\pi^*_{S2}$	3.92	0.68	4.81	9.41
Min-$^1\pi_{S2}\pi^*_{S2}$	3.65	0.95	5.90	10.5
Min-$^1n_{S2}\pi^*_{S2}$	3.17	1.43	6.23	10.83
			6.34	10.94
Min-$^3\pi_{S2}\pi^*_{S2}$	2.85	1.75	6.51	11.11
250 nm (4.9 eV)				
Min-$^1\pi_{S4}\pi^*_{S2}$	3.92	0.98	4.81	9.71
Min-$^1\pi_{S2}\pi^*_{S2}$	3.65	1.25	5.90	10.8
Min-$^1n_{S2}\pi^*_{S2}$	3.17	1.73	6.23	11.13
			6.34	11.24
Min-$^3\pi_{S2}\pi^*_{S2}$	2.85	2.05	6.51	11.41

It should be noted that the calculated photoelectron binding energies shown in Figure 4 (main text) for different states represent a $\Delta v = 0$ propensity during photoionization, *i.e.*, the transfer of vibrational excitation into the cation, as a simplistic way to estimate the photoelectron band maxima. For the case of ionization into the vibrationless cation, the predicted binding energy lies within the observation window, even for EAS2 and EAS3. This is explicitly shown in the figure below for 361 nm as an example. For each EAS, the circle marks the calculated electron binding energy to the vibrationless cation, and the arrow tip points to the expected band maximum (Table S2, last column).

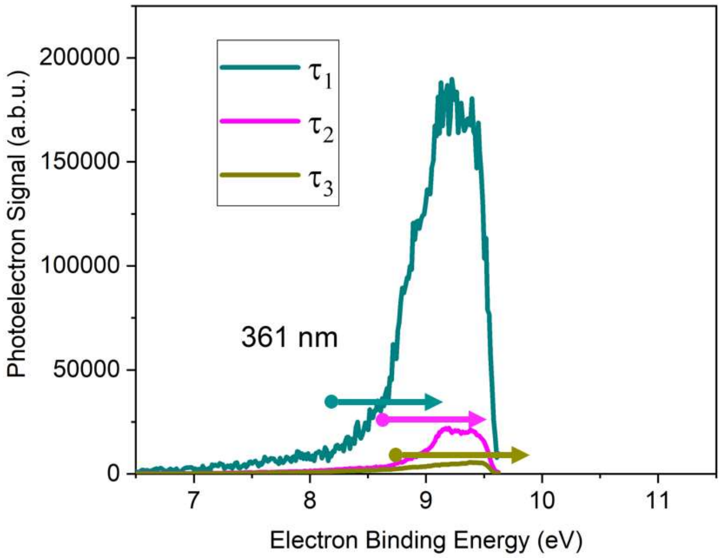

Figure S9. The EAS of 361 nm was extracted from a global lifetime analysis of the experimental data. The figure serves as an example of the range where the photoelectron signal is expected to rise. The arrow marks the energies required for ionization from the vibrationally hot excited state minimum to the vibrationless cation (circle) and the transfer of all excited state vibrational energy into the cation (tip). The latter is assumed to provide a rough approximation for the photoelectron band maximum.

The normalized EASs associated with different decays are replotted to facilitate a direct comparison of wavelength-dependent shifts and changes in spectral features. In Figure S10, the normalized EASs are grouped and replotted in three graphs according to their associated decays. The plots in panel b) visualize a wavelength-dependent shift in the EASs. As the excitation energy increases, the additional photon energy is converted into vibrational excitation during the electronic relaxation process. The observed wavelength-dependent shifts of the EASs resemble the differences in photon energy. In panel a), all EASs have been shifted in energy to overlap and enable direct comparison of spectral features. As higher states are photoexcited, changes in the shape of the photoelectron bands become evident for the τ_1-EASs. In the main manuscript, the lower energy feature has been associated with ionization from the $\pi_{S4}\pi^*_{S2}$ state, while the higher band corresponds to the $\pi_{S2}\pi^*_{S2}$.

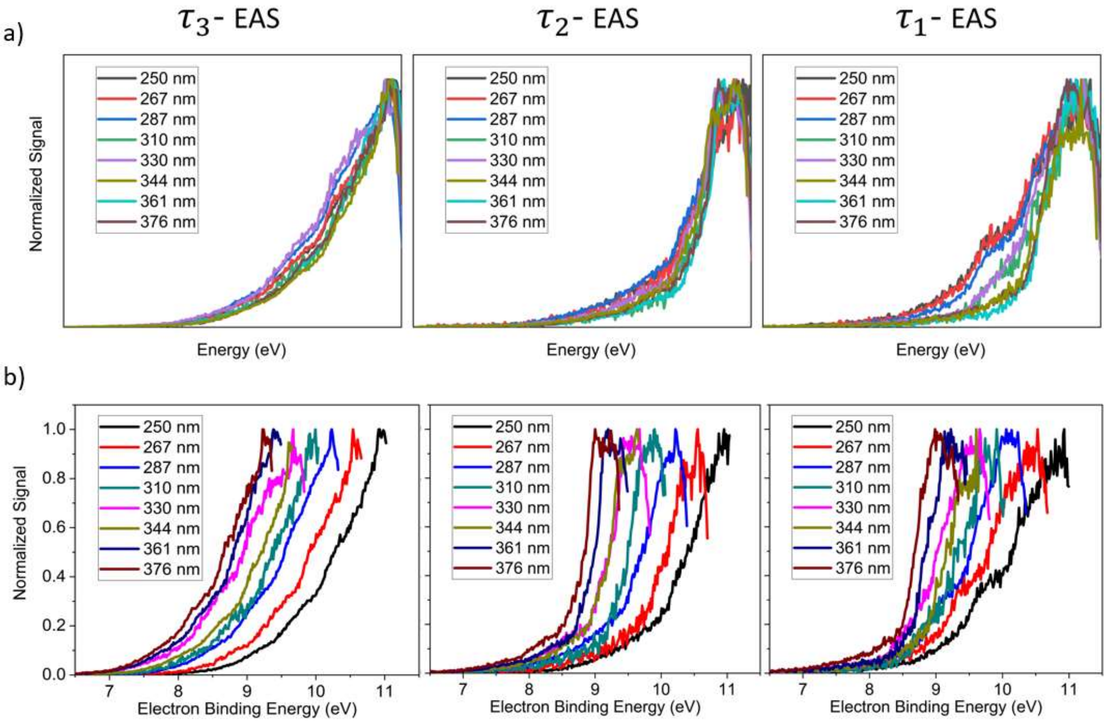

Figure S10. Normalized experimental EAS of all wavelengths are directly compared for each decay channel. Panel a) compares the EAS spectra for the three decay steps while all the spectra are manually shifted on the energy axis to overlap and highlight any changes in the shape or features of the spectrum. Panel b) compares the normalized spectra based on their energy axis to highlight the shift in the photoelectron spectrum due to vibrational excitation at higher pump photon energies.

S6. Detailed Decay Paths

The following Figure S11 is a more detailed version of Figure 3 in the main text, which includes additional critical points and state energies not directly discussed in the main text.

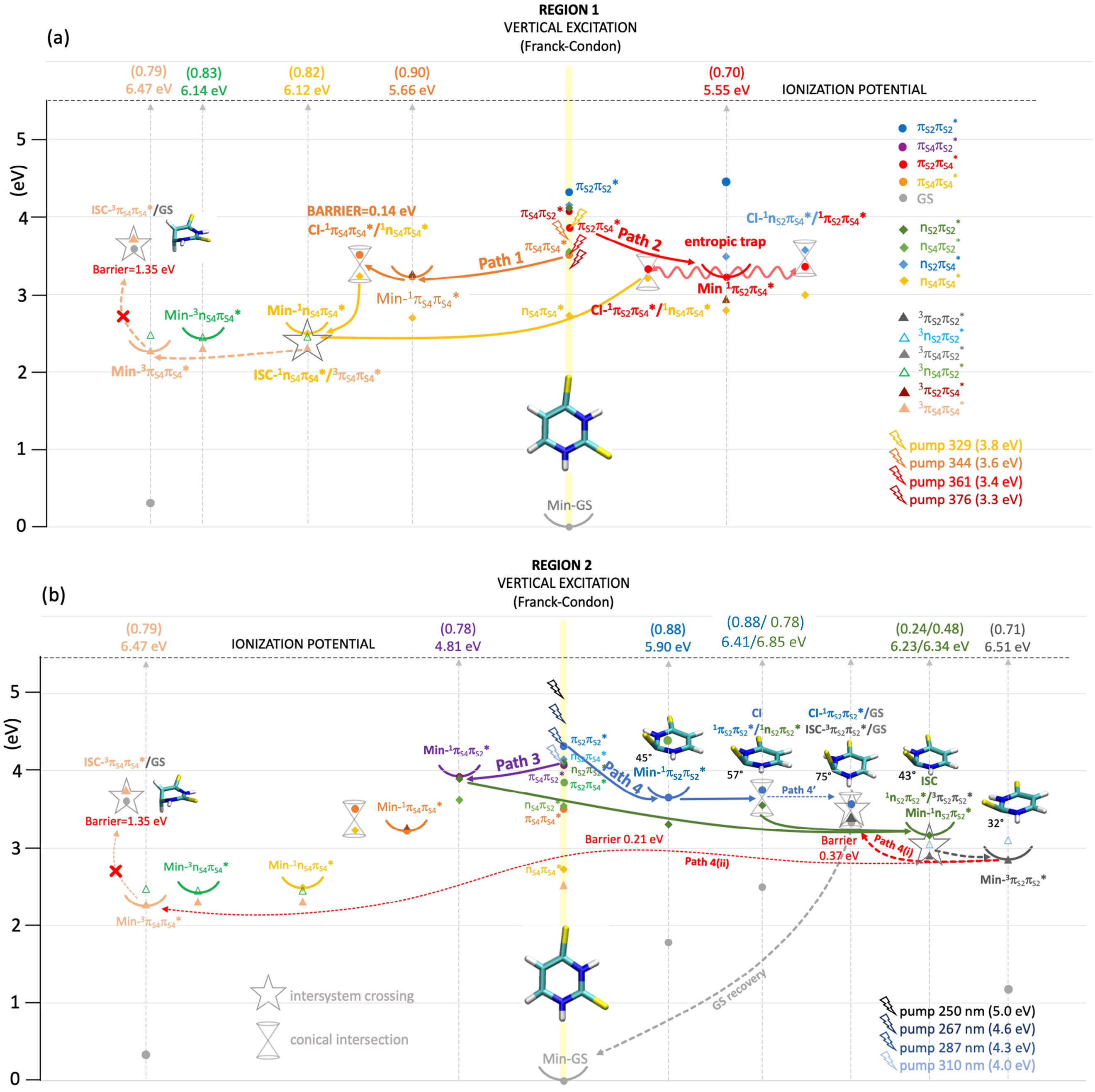

S7. Water Solvent Effect on Triplet Minima and Intersystem Crossings

From the perspective of potential applications, extending the detailed mechanistic picture from the gas phase to other environments is important. While the derived knowledge is directly transferable to non-polar or weakly interacting surroundings, its validity in polar environments is evaluated here. Additional calculations were performed to verify characteristics of the triplet potential are preserved in a polar solvent. Specifically, the two triplet minima documented in Figure 3 (Min-$^3\pi_{S4}\pi_{S4}$* and Min-$^3\pi_{S2}\pi_{S2}$*) and the two corresponding ISCs between the triplet and ground state (ISC-$^3\pi_{S4}\pi_{S4}$*/GS and ISC-$^3\pi_{S2}\pi_{S2}$*/GS), were optimized at MS-CASPT2/ANO-RCC level within a Polarizable Continuum Model[3-5] representing an aqueous environment. The results are visualized schematically in Figure S12 below.

The energy barriers and the molecular distortions computed for the solution phase closely resemble those under isolated conditions. This leads to the conclusion that the excitation wavelength-dependent photodynamics, and hence the tunability of the 2,4 DTU photoresponse, will be preserved across different environments.

For example, for excitation in the UVA region, the calculated energy gap between the Min-$^3\pi_{S4}\pi_{S4}$* and the ISC-$^3\pi_{S4}\pi_{S4}$*/GS is 1.03 eV in water (1.35 eV in the gas phase). This absence of an accessible decay funnel for GS recovery is characteristic of 4-TU and results in extended triplet lifetimes on the order of ns-μs timescales.

On the other hand, the triplet minimum, Min-$^3\pi_{S2}\pi_{S2}$*, populated via excitation in the UVB region, shows the same Sulfur 2 out-of-plane distortion in water as observed in the gas phase, and the energy barrier to the ISC-$^3\pi_{S2}\pi_{S2}$*/GS slightly increased (0.45 eV in water versus 0.37 eV in the gas phase). While this will still facilitate fast GS recovery, at the same time, it explains the longer triplet lifetimes observed in a solvent. This was documented in side-by-side solution- and gas-phase experiments that directly compared the wavelength-dependent dynamics of 2-TU.[6]

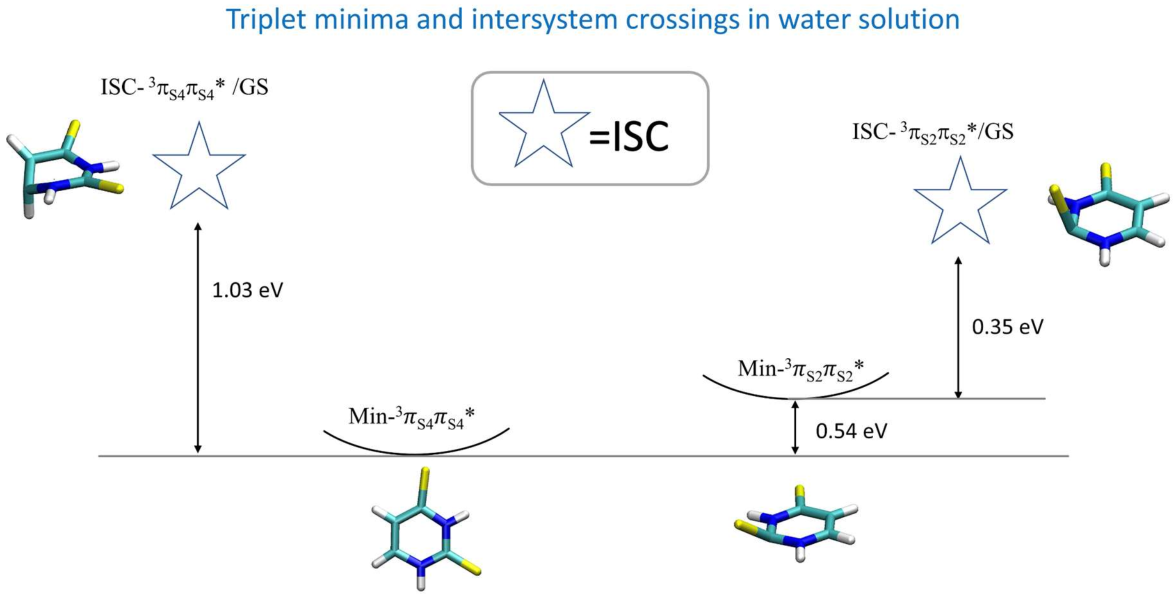

Figure S12: MS-CASPT2(18,14)/ANO-RCC energies and structures calculated in water solution Polarizable Continuum Model). The cartesian coordinates of the optimized structures in water are presented in section S9.

S8. Comparison of 2-TU, 4-TU and 2,4-DTU Decay Constants

As described in the main text, 2,4-DTU follows kinetics similar to 2-TU when excited in the UVB region of the absorption spectrum and 4-TU when excited in the UVA region of the absorption spectrum. The ISC and triplet state lifetimes of 2,4-DTU mirror this similarity to the singly substituted thiouracils. The table below directly compares 2-TU, 4-TU, and 2,4-DTU dynamics fitted with the same sequential exponential model.

The similarity in the triplet state lifetime is evident. Considering the negligible energy barrier for the ISC from $^1n\pi^*$ to $^3\pi\pi^*$ for both 4-TU and 2,4-DTU (excited in region 1), the 626fs and 2660fs might seem to differ more than expected. Nevertheless, these ISC dynamics are slower compared to 2-TU or 2,4-DTU-excited in region 2. The generally faster ISC in 2,4-DTU could stem from higher SOC because the two sulfur atoms increase the coupling to the triplet state. This was also observed computationally, where this SOC is calculated 223 cm^{-1} for 2,4-DTU and 160 cm^{-1} for 4-TU.[7]

Table S3. 2,4-DTU decay constants, excited in the UVA and UVB region of the absorption spectrum, are compared with the decay constants of 4-TU[8] and 2-TU[6]. The 2,4-DTU, photoexcited at 361nm (yellow highlighted row), follows relatively similar dynamics as 4-TU (third row, also highlighted with yellow), and it follows the 2-TU dynamics (last row, highlighted with blue) when excited at 267nm (second row, highlighted with blue color).

Pump wavelength (nm)	Molecule	τ_1 (fs)	τ_2 (fs)	τ_3 (ns)	Ref.
361	2,4-DTU	30 ± 25	626 ± 50	> 2	[1]
267	2,4-DTU	40 ± 20	240 ± 20	0.54	This work
330	4-TU	< 40	2660 ± 250	> 1.5	[8]
260	2-TU	67 ± 15	285 ± 60	0.856	[6]

S9. Cartesian Coordinates

Cartesian Coordinates in water solution:

Min-$^3\pi_{S4}\pi_{S4}$* (in water)

C	17.677169	16.418450	14.679547
N	18.101555	17.622189	14.302086
C	18.461276	18.641602	15.186068
C	18.355297	18.378522	16.561297
C	17.920133	17.151628	16.971472
N	17.594440	16.193755	15.991806
H	18.612837	19.132317	17.276033
S	17.700922	16.680756	18.652414
S	17.261068	15.225547	13.550588
H	18.802164	19.556889	14.757344
H	17.285715	15.288071	16.281481
H	18.156635	17.792105	13.318300

Min-$^3\pi_{S2}\pi_{S2}$* (in water)

C	17.615269	16.365428	14.671509
N	18.043022	17.633610	14.300528
C	18.380045	18.570254	15.203333
C	18.338578	18.343645	16.528468
C	17.894152	17.063499	16.988898
N	17.579512	16.163908	16.057156
H	18.614866	19.095959	17.232955
S	17.755474	16.669217	18.645076
S	18.346381	15.065110	13.654140
H	18.679988	19.516952	14.795249
H	17.264924	15.266462	16.373327
H	17.999480	17.881400	13.331193

ISC-$^1n_{S4}\pi_{S4}$*/$^3\pi_{S4}\pi_{S4}$* (in water)

C	17.623463	16.567611	14.688596
N	17.746278	17.853640	14.350325
C	18.626154	18.614827	15.167575
C	18.190515	18.494160	16.573961
C	18.056493	17.159250	17.003100
N	18.021971	16.235189	15.943675
H	17.665788	19.280774	17.083661
S	17.770921	16.674335	18.556367
S	17.040601	15.397183	13.631748
H	19.675185	18.449541	14.956026
H	17.910412	15.271133	16.185849
H	17.601431	18.084187	13.387554

ISC-$^3\pi_{S2}\pi_{S2}$*/GS (in water)

C	17.439272	16.415610	14.574298
N	17.834395	17.720991	14.275154
C	18.364353	18.561990	15.178735
C	18.444828	18.265054	16.489114
C	17.913446	17.018257	16.932075
N	17.466788	16.178350	15.969226
H	18.848593	18.954194	17.196650
S	17.791422	16.577243	18.546299
S	18.918575	15.084819	14.223934
H	18.705357	19.504831	14.792708
H	17.004917	15.346882	16.284170
H	17.779802	18.007276	13.31951

Cartesian Coordinates in the gas phase:

Min-GS

C	17.758354	16.301690	14.598135
N	18.130070	17.580152	14.275097
C	18.366011	18.554378	15.194636
C	18.269506	18.304836	16.525052
C	17.939166	16.986357	16.972414
N	17.667198	16.092446	15.956168
H	18.481511	19.069817	17.254354
S	17.880059	16.522077	18.559449
S	17.456355	15.140619	13.473168
H	18.647683	19.517148	14.792844
H	17.370338	15.157706	16.223042
H	18.239196	17.789446	13.286981

Min-$\pi_{S4}\pi_{S4}^*$

C	17.778603	16.344599	14.622560
N	18.127171	17.632289	14.299664
C	18.372627	18.660741	15.153077
C	18.270293	18.381385	16.571843
C	17.926000	17.125816	16.942378
N	17.680231	16.150952	16.000339
H	18.473445	19.143081	17.304525
S	17.748815	16.444369	18.613576
S	17.511654	15.155223	13.502297
H	18.625011	19.612764	14.719401
H	17.403267	15.233756	16.326696
H	18.196353	17.781634	13.300658

CI-$\pi_{S4}\pi^*_{S4}/n_{S4}\pi^*_{S4}$

C	17.737848	16.359098	14.618990
N	18.115850	17.644198	14.299413
C	18.355713	18.680086	15.110658
C	18.193296	18.409126	16.586444
C	17.954000	17.139038	16.917862
N	17.749937	16.148876	16.009476
H	18.302203	19.192156	17.314490
S	17.726405	16.333955	18.631020
S	17.360140	15.210405	13.483682
H	18.829874	19.546574	14.677335
H	17.577537	15.231317	16.411767
H	18.210669	17.771782	13.295876

Min-$n_{S4}\pi^*_{S4}$

C	17.728704	16.354585	14.631569
N	18.128609	17.610462	14.283145
C	18.417123	18.637581	15.187231
C	18.315129	18.376203	16.541801
C	17.925050	17.113002	16.958579
N	17.634717	16.146302	15.981008
H	18.535847	19.149327	17.262301
S	17.780692	16.584333	18.617714
S	17.375733	15.167420	13.528317
H	18.706396	19.586514	14.772441
H	17.338574	15.218558	16.242168
H	18.193806	17.765957	13.291360

Min-$^3n_{S4}\pi^*_{S4}$

C	17.778139	16.346982	14.630725
N	18.128692	17.618563	14.289886
C	18.378753	18.647971	15.202474
C	18.273229	18.374750	16.553674
C	17.923127	17.094159	16.964822
N	17.694588	16.119707	15.980270
H	18.459583	19.149019	17.280817
S	17.762044	16.580508	18.618262
S	17.477818	15.157474	13.515514
H	18.634833	19.607788	14.790615
H	17.413512	15.184857	16.231196
H	18.189152	17.784832	13.298758

Min-$^{3}\pi_{S4}\pi_{S4}^{*}$

C	17.667164	16.388291	14.657604
N	18.110210	17.637670	14.301100
C	18.446871	18.657300	15.180997
C	18.353581	18.381890	16.559072
C	17.916272	17.146553	16.982049
N	17.569530	16.195148	16.007448
H	18.623641	19.127581	17.288955
S	17.739558	16.674248	18.644447
S	17.285943	15.225397	13.541467
H	18.791126	19.582576	14.755640
H	17.242653	15.288060	16.304298
H	18.182726	17.777176	13.305412

ISC-$^{3}\pi_{S4}\pi_{S4}^{*}$/GS

C	17.619912	16.554861	14.683199
N	17.734387	17.862023	14.356879
C	18.621088	18.616216	15.163135
C	18.203455	18.489121	16.575915
C	18.051656	17.156462	17.004660
N	18.032784	16.233800	15.945480
H	17.688574	19.275495	17.086672
S	17.748459	16.676109	18.559862
S	17.056824	15.405424	13.630888
H	19.668748	18.464732	14.941827
H	17.903967	15.278443	16.184515
H	17.599414	18.069198	13.395451

Min-$\pi_{S2}\pi_{S4}^{*}$

C	17.782333	16.383599	14.670894
N	18.134381	17.615677	14.268797
C	18.371753	18.668577	15.187825
C	18.265494	18.366662	16.542152
C	17.926294	17.092938	17.040911
N	17.663262	16.134888	15.969380
H	18.460745	19.143946	17.266306
S	17.783525	16.511310	18.607879
S	17.496509	15.162411	13.494176
H	18.605331	19.627264	14.763668
H	17.392689	15.205720	16.269552
H	18.231158	17.753618	13.275473

CI-$\pi_{S2}\pi_{S4}^{*}/n_{S4}\pi_{S4}^{*}$

C	17.779587	16.406854	14.692369
N	18.123853	17.613434	14.256572
C	18.376669	18.679599	15.181846
C	18.260576	18.356734	16.540137
C	17.924873	17.088842	17.087498
N	17.664871	16.134473	15.962837
H	18.453192	19.139114	17.259162
S	17.772694	16.495897	18.605535
S	17.507284	15.173058	13.469953
H	18.629892	19.629558	14.748818
H	17.407017	15.207727	16.293283
H	18.212965	17.741320	13.259004

CI-$\pi_{S2}\pi_{S4}^{*}/n_{S2}\pi_{S4}^{*}$

C	17.792408	16.409263	14.687199
N	18.116818	17.597035	14.279435
C	18.376425	18.652210	15.202454
C	18.268408	18.359056	16.544199
C	17.928967	17.116540	17.006895
N	17.694305	16.145629	15.950755
H	18.456211	19.129102	17.261388
S	17.735271	16.512788	18.570875
S	17.482929	15.145185	13.507696
H	18.629831	19.590325	14.770563
H	17.445415	15.237382	16.268193
H	18.186483	17.772093	13.307361

Min-$\pi_{S4}\pi_{S2}^{*}$

C	17.766962	16.308640	14.601050
N	18.131105	17.661957	14.297488
C	18.365633	18.615645	15.183774
C	18.265188	18.362313	16.554742
C	17.902251	17.044737	16.932579
N	17.679811	16.132225	16.001364
H	18.454276	19.114638	17.300455
S	17.790739	16.662243	18.663663
S	17.490859	15.142099	13.435993
H	18.636371	19.586372	14.790978
H	17.425355	15.192473	16.290999
H	18.204923	17.843268	13.303928

Min-$\pi_{S2}\pi_{S2}^{*}$

C	17.407799	16.454489	14.637111
N	17.942897	17.679131	14.279709
C	18.382159	18.601411	15.189577
C	18.356365	18.327623	16.522952
C	17.901383	17.043439	16.996737
N	17.561351	16.167205	15.979603
H	18.686101	19.056122	17.244908
S	17.772432	16.601993	18.584770
S	18.112924	15.090197	13.585806
H	18.755794	19.530147	14.787261
H	17.294426	15.229228	16.258616
H	17.939842	17.885624	13.289963

CI-$\pi_{S2}\pi_{S2}^{*}/n_{S2}\pi_{S2}^{*}$

C	17.305895	16.487913	14.637760
N	17.864658	17.692232	14.285606
C	18.357150	18.600539	15.181353
C	18.354422	18.316479	16.512909
C	17.887714	17.042439	16.994720
N	17.468966	16.204722	15.967995
H	18.756725	19.016640	17.226418
S	17.852106	16.553973	18.571414
S	18.313760	15.168328	13.706178
H	18.769509	19.509072	14.770129
H	17.229239	15.248280	16.214771
H	17.953326	17.825992	13.287760

Min-$n_{S2}\pi_{S2}^{*}$ (ISC-nπ_{S2}^{*}/$^{3}\pi_{S2}\pi_{S2}^{*}$)

C	17.525409	16.401976	14.610650
N	17.973443	17.675776	14.259635
C	18.378445	18.595491	15.186740
C	18.379968	18.316092	16.518884
C	17.914181	17.030771	16.981523
N	17.529218	16.159087	15.991146
H	18.703089	19.041847	17.245064
S	17.808987	16.595982	18.579826
S	18.142938	15.081044	13.588225
H	18.682756	19.552834	14.791069
H	17.160273	15.275523	16.313510
H	17.914765	17.940185	13.290741

Min-$^3\pi_{S2}\pi^*_{S2}$

C	17.466473	16.432396	14.625964
N	18.030883	17.658113	14.278184
C	18.383660	18.601278	15.187429
C	18.297941	18.347696	16.530930
C	17.902985	17.039363	16.996078
N	17.586870	16.152520	15.996313
H	18.571632	19.093112	17.257406
S	17.857915	16.595245	18.591336
S	17.909438	15.117164	13.519127
H	18.722570	19.545309	14.790232
H	17.304724	15.228522	16.291646
H	18.078380	17.855893	13.292366

CI-$\pi_{S2}\pi^*_{S2}$/GS and ISC-$^3\pi_{S2}\pi^*_{S2}$/GS

C	17.450197	16.416535	14.555883
N	17.921703	17.703985	14.255302
C	18.350456	18.601713	15.184561
C	18.398602	18.306814	16.515289
C	17.958866	17.020535	16.972717
N	17.579468	16.136857	15.961390
H	18.728493	19.033458	17.237772
S	17.831667	16.550141	18.554443
S	18.586500	15.040146	14.175313
H	18.647980	19.566575	14.801139
H	17.090037	15.311775	16.287820
H	17.967783	17.946971	13.280255

S10. References

(1) Mohamadzade, A.; Ullrich, S. Internal conversion and intersystem crossing dynamics of uracil upon double thionation: a time-resolved photoelectron spectroscopy study in the gas phase. *Physical Chemistry Chemical Physics* **2020**, *22* (27), 15608-15615.

(2) Ruckenbauer, M.; Mai, S.; Marquetand, P.; González, L. Photoelectron spectra of 2-thiouracil, 4-thiouracil, and 2, 4-dithiouracil. *The Journal of Chemical Physics* **2016**, *144* (7), 074303.

(3) Barone, V.; Cossi, M. Quantum calculation of molecular energies and energy gradients in solution by a conductor solvent model. *The Journal of Physical Chemistry A* **1998**, *102* (11), 1995-2001.

(4) Cossi, M.; Barone, V. Solvent effect on vertical electronic transitions by the polarizable continuum model. *The Journal of Chemical Physics* **2000**, *112* (5), 2427-2435.

(5) Cossi, M.; Rega, N.; Scalmani, G.; Barone, V. Polarizable dielectric model of solvation with inclusion of charge penetration effects. *The Journal of Chemical Physics* **2001**, *114* (13), 5691-5701.

(6) Sánchez-Rodríguez, J. A.; Mohamadzade, A.; Mai, S.; Ashwood, B.; Pollum, M.; Marquetand, P.; González, L.; Crespo-Hernández, C. E.; Ullrich, S. 2-Thiouracil intersystem crossing photodynamics studied by wavelength-dependent photoelectron and transient absorption spectroscopies. *Physical Chemistry Chemical Physics* **2017**, *19* (30), 19756-19766.

(7) Borrego-Varillas, R.; Teles-Ferreira, D. C.; Nenov, A.; Conti, I.; Ganzer, L.; Manzoni, C.; Garavelli, M.; Maria de Paula, A.; Cerullo, G. Observation of the sub-100 femtosecond population of a dark state in a thiobase mediating intersystem crossing. *Journal of the American Chemical Society* **2018**, *140* (47), 16087-16093.

(8) Mohamadzade, A.; Bai, S.; Barbatti, M.; Ullrich, S. Intersystem crossing dynamics in singly substituted thiouracil studied by time-resolved photoelectron spectroscopy: Micro-environmental effects due to sulfur position. *Chemical Physics* **2018**, *515*, 572-579.
